# Multiple ultradian rhythms of metabolism, body temperature and activity in Djungarian hamsters

**DOI:** 10.1007/s00360-024-01569-x

**Published:** 2024-07-05

**Authors:** Gerhard Heldmaier, Luzie Braulke, Johanna Flick, Thomas Ruf

**Affiliations:** 1 Animal Physiology, Department of Biology, Marburg University, Karl-von-Frisch Str. 8, 35032 Marburg, Germany; 2 Institute of Wildlife Ecology, University of Veterinary Medicine, Vienna, Austria

**Keywords:** Wavelet analysis, Metabolic rate, Bioenergetics, Calorimetry, Infrared thermovision

## Abstract

Djungarian hamsters *(Phodopus sungorus)* living at constant 15 °C T_a_ in short photoperiod (8:16 h L:D) showed pronounced ultradian rhythms (URs) of metabolic rate (MR), body temperature (T_b_) and locomotor activity. The ultradian patterns differed between individuals and varied over time. The period length of URs for MR, T_b_ and activity was similar although not identical. Wavelet analysis showed that three different URs are existing in parallel, URs of small amplitude and short duration (UR*small*), URs of medium amplitude and medium duration (UR*medium*) and URs of large amplitude (UR*large*), superimposed on each other. UR*large* were accompanied by an increase in locomotor activity, whereas UR*small* and UR*medium* were of metabolic origin with lacking or delayed responses of activity. An energetic challenge to cold which raised total energy requirements by about 50% did not accelerate the period length of URs, but extended the amplitude of UR*small* and UR*medium*. UR*large* corresponds with the URs of activity, feeding and drinking, sleep and arousal as described in previous studies, which are related to midbrain dopaminergic signalling and hypothalamic ultradian signalling. The cause and control of UR*medium* and UR*small* is unknown. Their periods are similar to periods of central and peripheral endocrine ultradian signalling, suggesting a link with URs of metabolism.

## Introduction

Ultradian rhythms (URs) were first discovered in locomotor activity patterns of small mammals (Aschoff and Gerkema 1985; Honma and Hiroshige 1978; Daan and Aschoff 1981). Their real nature was a mystery largely because of their variability and broad frequency range with periods lasting between 20 min and 6 h, quite in contrast to the narrow bandwidth of the circadian rhythms close to 24 h. Research in the past 40 years disclosed that URs are occurring in all animals and even in single cells. They are generated by cellular ultradian oscillators including translation-transcription feedback loops coordinating time dependent cellular processes (Isomura and Kageyama 2014; Goh et al. 2019). URs are coupled between cells, which was recently observed as a gap-junction mediated process in confluent fibroblast cell cultures thus allowing coordinated activities of groups of cells or tissues (Yang et al. 2022).

URs are centrally controlled by the hypothalamus suggesting a leading role of the paraventricular nucleus (PVN). In voles, dissections of the PVN suppressed the expression of URs, while dissections of the SCN had no effect on URs but abolished circadian rhythms (CR) (Gerkema et al. 1990b). Ultradian calcium rhythms measured in hypothalamic brain slices in vitro revealed a similar constellation, i.e. calcium URs were generated in the PVN and the subparaventricular zone (SPZ) but not in the SCN (Wu et al. 2018). The latter showed ultradian properties but only when connected with the PVN-SPZ. An additional pathway of central control is linked to the brain dopamine level. In mice the dopamine level in the striatum fluctuates with the ultradian pattern of locomotor activity. A knockout of the dopamine transporter prolongs the ultradian activity period from 4 to 12 h. This suggests the existence of a dopaminergic ultradian oscillator (DUO) for ultradian rhythms of behavioural arousal (Blum et al. 2014; Kashiwagi et al. 2021).

The SCN is considered as the master clock of the circadian rhythm (CR) which establishes internal synchronisation with the circadian oscillators in peripheral cells and tissues as well as external synchronisation with the light dark cycle (reviews in Mohawk et al. 2012; Schibler et al 2015). Synchronisation with the external light dark signal explains why the circadian clock mechanism has evolved for high precision at a period of 24 h. This need is lacking for URs and explains why they can vary over a broad frequency range from 20 min to 6 h. There is some coordination between the CR and UR (Aschoff and Gerkema 1985, Ono et al. 2015), however URs continue in the absence of external cues as well as following SCN ablation and the concomitant loss of circadian changes in locomotor activity and T_b_ (Honma and Hiroshige 1978, Gerkema et al. 1990b).

URs are reflecting time dependent and episodic metabolic processes in cells, tissues and the entire organism. Their coordination is essential for metabolic pathway organisation as well as for the amplitude changes of metabolic rate (MR) required for adequate adjustments of energy supply for locomotion, heat production etc. (Ootsuoka et al. 2009, Blessing 2012, Meyer et al. 2012; Grant et al. 2018). There can also be a feedback of environmental conditions on the expression of ultradian rhythms, e.g. food shortage enhances ultradian rhythms in voles (van Rosmalen and Hut 2021).

URs of animals were demonstrated mostly for activity behaviour, feeding, neural and endocrine rhythms. Little attention was paid to URs of metabolic rate and other metabolic parameters, largely because of technical limitations for measuring animal metabolism in vivo at high resolution over prolonged periods of time. Brodsky (2014) has emphasized that circahoralian (ultradian) rhythms of metabolic processes like protein biosynthesis, cell respiration and the mitotic cell cycle occur in parallel with ultradian rhythms of endocrines, suggesting that endocrine signalling plays an important role in the control of cellular ultradian rhythms. Hormones which are known for direct effects on metabolism of whole animals, like catecholamines, thyroid hormones and glucocorticoids, may therefore also play an important role in the expression of ultradian metabolic processes on the whole animal level.

We recorded metabolic rate and respiratory exchange rate of Djungarian hamsters at 1 min intervals continuously over several months in parallel with telemetry of T_b_ and locomotor activity to obtain a detailed picture of stability and changes of URs in all four parameters, and to analyse the role of ultradian rhythms for the control of metabolic rate.

## Methods

### Animals

Djungarian Hamsters (*Phodopus sungorus*) were bred and raised at the Department of Biology at Marburg University, as described previously (Ruf et al. 1993). They received food (Ssniff V2140-000) and water ad libitum. At the age of three months they were kept singly in standard Makrolon cages, Type 3, and transferred from long photoperiod (L:D 16:8 h) and 23 ℃ T_a_ to short photoperiod (L:D 8:16 h) at 23 °C T_a_. Body mass and fur colour index were recorded at weekly intervals to follow acclimation to short photoperiod.

Fifteen hamsters of both sexes were used in this study. They were kept in metabolic cages for two months for continuous records of MR (see also Heldmaier et al. 2024). Eight of these hamsters were additionally implanted with transmitters for T_b_ and locomotor activity for the simultaneous measurement of MR, Tb, and activity and the analysis of ultradian rhythms. Seven of these hamsters received transmitters for T_b_ only and were used for the energy challenge experiments. Description of methods see below.

For measurement of MR the hamsters were transferred to ventilated cages (Zoonlab, Castrop-Rauxel, Germany, IVC cages, Type 1 long (volume 8 L)), with little bedding material (wood shavings 80 g) and two paper towels, which they gnawed to build a nest. One separate cage setup (glass terrarium, volume 12 L) was equipped with a video camera and an IR-thermovision camera (Optris PI450, Optris GmbH, Berlin) for additional continuous observation of hamster behaviour and measurement of surface temperature. Ambient temperature of the climate chamber was controlled at 14 ± 1 °C and was recorded for each hamster by a thermocouple inside the cage. Cages were cleaned and bedding material renewed at weekly intervals. For the latter measurements 4 out of the 8 hamsters were transferred for at least 10 days to the glass terrarium and then returned to their IVC cage.

### Transmitters for T_b_ and locomotor activity

Transmitters for simultaneous recording of T_b_ and activity (Vitalview 4000, Starr Life Sciences, Oakmont, PA, USA) were implanted with the same procedure as described previously for other abdominal transmitters (e.g. Braulke et al. 2008). Briefly, the hamsters were initially anaesthetized with Rompun (1 mg kg^−1^) and Ketanest (50 mg kg^−1^). Rompun was used as an analgesic. During surgery anaesthesia was controlled and maintained with Isoflurane. The transmitter was implanted into the abdominal cavity without fixation. The wound was closed with an abdominal suture plus a skin suture. Typically, the hamsters aroused from anaesthesia within 20 min, walked around and started drinking and feeding, and were left for 5 days in their cage for full recovery. T_b_ (℃) and activity (counts min^−1^) was recorded in 1 min intervals during the entire measurement sessions lasting up to three months.

### Behaviour and locomotion by infrared thermovision

Behaviour and locomotor activity were further analysed by an infrared camera system (Optris PI 450i, Optris GmbH, Berlin) which was mounted on the top of the cage. This auto-calibrated thermovision camera measured surface temperatures (T_sf_) with a spatial resolution of 382 × 288 pixels and a thermal resolution of 0.04 K. T_sf_ images were continuously recorded and stored at 1 s intervals. For analysis we specified three measurement areas in the T_sf_ images. One area traced the hamster, following it at any position in the cage, within its nest or outside the nest, during feeding and drinking. Two fixed measurement areas monitored the bottom of the cage outside the nest. This allowed us to locate the position of the hamster, its T_sf_ and the surrounding temperatures. Hamster T_sf_’s were always higher than the cage bottom, and T_sf_ of eyes and nostrils were close to core temperature of the hamster. Body movements caused short term changes of T_sf_. Standard deviation (SD) of T_sf_ min^−1^was calculated as an index for total activity of the hamsters.

### Metabolic rate

Metabolic rate (mL O_2_ min^−1^) was measured with CaloBoxes (Phenosys GmbH, Berlin, Germany) which were directly connected to the lids of ventilated cages (Zoonlab GmbH, IVC type 1 long, Castrop-Rauxel, Germany) (Elfers et al. 2022). We used up to 8 CaloBoxes simultaneously to obtain continuous records of individual hamsters. The CaloBoxes were placed near the animal cages in the climate chamber. Sample air was drawn from the animal cage with flow rates of ~ 85 L/h. Ambient air of the climate chamber was used as reference air. To buffer short term variations of climate chamber air conditions air supply for the animal cages and reference air were drawn from a 100 L reservoir (dust bin) inside the climate chamber.

The CaloBox measured O_2_, CO_2_, and water vapor content every 4 s and calculated RER and heat production. Heat production (HP) was calculated by using the equation HP[mW] = (4.44 + 1.43* RER)* [mLO_2_ h^−1^] which provides the oxidative energy retrieved from mixed combustion of carbohydrates and lipids (Heldmaier 1975). Zero adjustment and O_2_-sensor calibration with reference air occurred every 15 min. The CaloBox did not need any air processing (drying, CO_2_ absorption) prior to analysis but measured the composition of incoming air. Results were collected and stored every 30 s or 60 s (further details see Elfers et al. 2022; Wellbrock et al. 2022).

### Response of ultradian rhythms to thermal challenge

To measure the effect of energetic challenges on URs, data from a previous study were reanalysed. In this study MR was measured with a different system using an O_2_ analyser (S3a, Applied Electrochemistry), and a CO_2_ analyser (Maihak) (method see Braulke et al. 2008, 2010). Air was dried with a freeze trap. Data were sampled at 3 or 4 min intervals.

### Identification of ultradian rhythms

Long-term simultaneous records of T_b_, activity, and metabolic rate were used to identify ultradian rhythms. Ultradian rhythms are rather variable unlike the stable circadian rhythms which are controlled by an endogenous clock. To identify regular structures in ultradian rhythms we performed a Wavelet analysis and calculated the power spectrum (R package WaveletComp (Roesch and Schmidbauer 2018)) in R 4.2.2 (R Core Team 2022)) of regular, individual records of metabolic rate, Tb and locomotor activity. The wavelet transform is a method for determining instantaneous periods with good resolution in both time and frequency. Unlike Fourier transformations, wavelets are not based on infinitive trigonometric functions but on short waves that decay quickly to zero. This is also true for the popular Morlet wavelet, a complex sine decaying on both sides of the centre, which is the underlying function used by WaveletComp. The principal operation performed is the local convolution of the stretched or squished wavelets along the time series, effectively computing similarities. Thus, wavelet transformation analyses localized variations of amplitude within a time series. The amplitude of ultradian rhythms was determined by searching for peak maxima using a search window adjusted to the ultradian periods retrieved for each hamster and each day. To make URs highly comparable we did not change the wavelet or its parameters for the analysis of different variables (i.e., Tb, MR, activity). For the same reason we used continuous wavelet transform for all variables, despite certain differences between discrete and continuous data (Leise et al. 2013).

In the R package WaveletComp the statistical significance of the power of peaks (amplitudes relative to noise) is computed by simulation (we used 150), i.e., the comparison of actual power with random noise at the same frequency. Here we called the function with method = ”shuffle” This randomly shuffles the original data instead of using white noise, to make sure that the mode of data sampling does not affect results (Nemec and Nemec 1985). The frequencies inspected here were in the ultradian range (0.3–6 h). Because we were not interested in circadian rhythms, we limited data to 24 h. Time series analysed were also restricted to 24 h to avoid mixing days with and without torpor (for general recommendations on data length see Leise and Harrington 2011). Time series were on a regular grid (n = 1440) at 1 min intervals.

For wavelet analysis output-graphs the number of colours was set to 250, and power ridges (upper bands of neighbouring values in the frequency domain) were plotted but not further considered. To further determine the highest powers in a periodogram we used function findpeaks in the package pracma (Borchers 2019) using default parameters.

We mainly preferred wavelet analysis over traditional Fourier and related MESA or Lomb-Scargle periodograms because they all have potentially large peaks at harmonics of 24 h (12 h, 8 h, 6 h, etc.), even when the measured signal involves no ultradian periods. Data folding methods such as the Chi^2^ periodogram repeat every short-term peak at its multiple (Ruf 1999). These traditional methods will not be useful in measuring the true period(s) of ultradian activity/TB/MR patterns (Leise 2013).  More appropriate are time–frequency methods, such as wavelet analysis “that can localize frequency estimates in time are more appropriate for analysis of ultradian periods and of fluctuations in the period” (Leise 2013).

### Statistical evaluation of results

Results are either presented as original data or mean values. For comparison between groups ANOVA was used with p < 0.05 as general level of significance. Due to the nature of data in most cases ANOVA of repeated measures was applied, and differences between groups were compared with the Holm-Sidak Method (Jandel SigmaPlot10). Non-parametric tests (Tukey test, Wilcoxon test) were applied for data with a non-normal distribution. Visual inspection of model residuals (qqnorm plots) in R, gave no reason to suspect a notable deviation from normality.

## Results

### Ultradian rhythms of metabolic rate, activity and body temperature

**Fig. 1:**
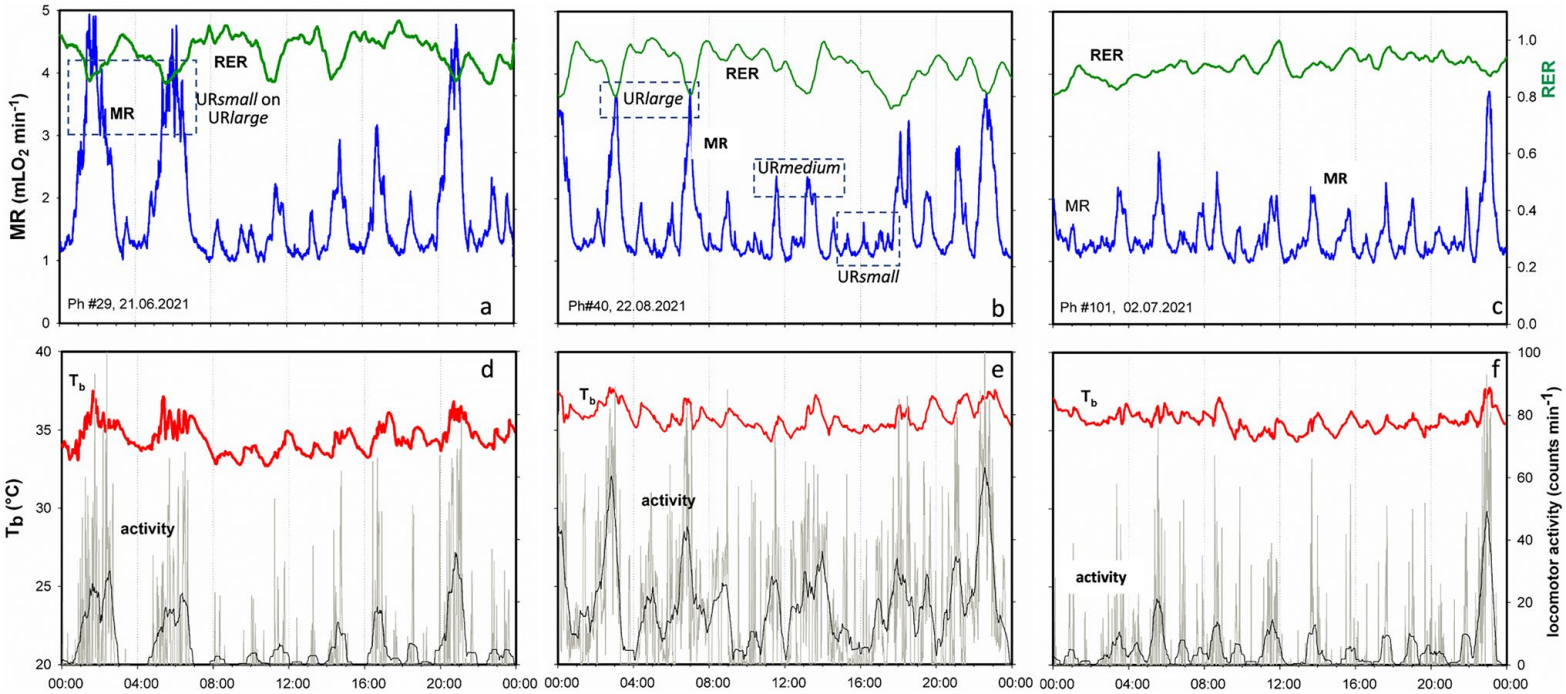
Ultradian rhythms of MR, RER, T_b_ and locomotor activity of short day acclimated Dungarian hamsters. Records of three different individuals and days are shown as examples for the individual variability of ultradian behaviour. Ph#29, male, M_b_ 33.9 g; Ph#40, female, M_b_ 32.5 g; Ph#101, male, M_b_ 31.2 g. Data were obtained in 1 min intervals. For locomotor activity raw data (gray) and 30 min running average (black) are shown. RER is presented as 30 min running average of raw data. URs of MR are characterized by at least three different amplitudes, i.e. UR*small*, UR*medium* and UR*large*

24 h records of metabolic rate, body temperature and locomotor activity of Djungarian hamsters kept in constant conditions were characterized by the presence of ultradian variations. The pattern varied between individuals but each hamster showed an apparently synchronous change in MR, T_b_ and bursts of locomotor activity (Fig. 1). The original record of MR revealed the existence of at least three different URs, which are existing simultaneously (Fig. 1). Most obvious were large amplitude fluctuations, where MR was > 100% above RMR (UR*large*). Small amplitude fluctuations about 20% above RMR were traceable throughout the entire day and are also found superimposed as peaks on UR*large*. In between these two extreme fluctuations of MR a third category of medium sized URs could be discriminated with an amplitude of about 50% above RMR (UR*medium*). Examples for these three different URs are framed in Fig. 1b, as well as superimposition of UR*small* on UR*large* shown in Fig. 1a. The 24hourly records of T_b_ and locomotor activity show URs with rather uniform amplitudes (Fig. 1 d, e, f) as compared to the varying amplitude of MR URs (Fig. 1a, b, c).

**Fig. 2:**
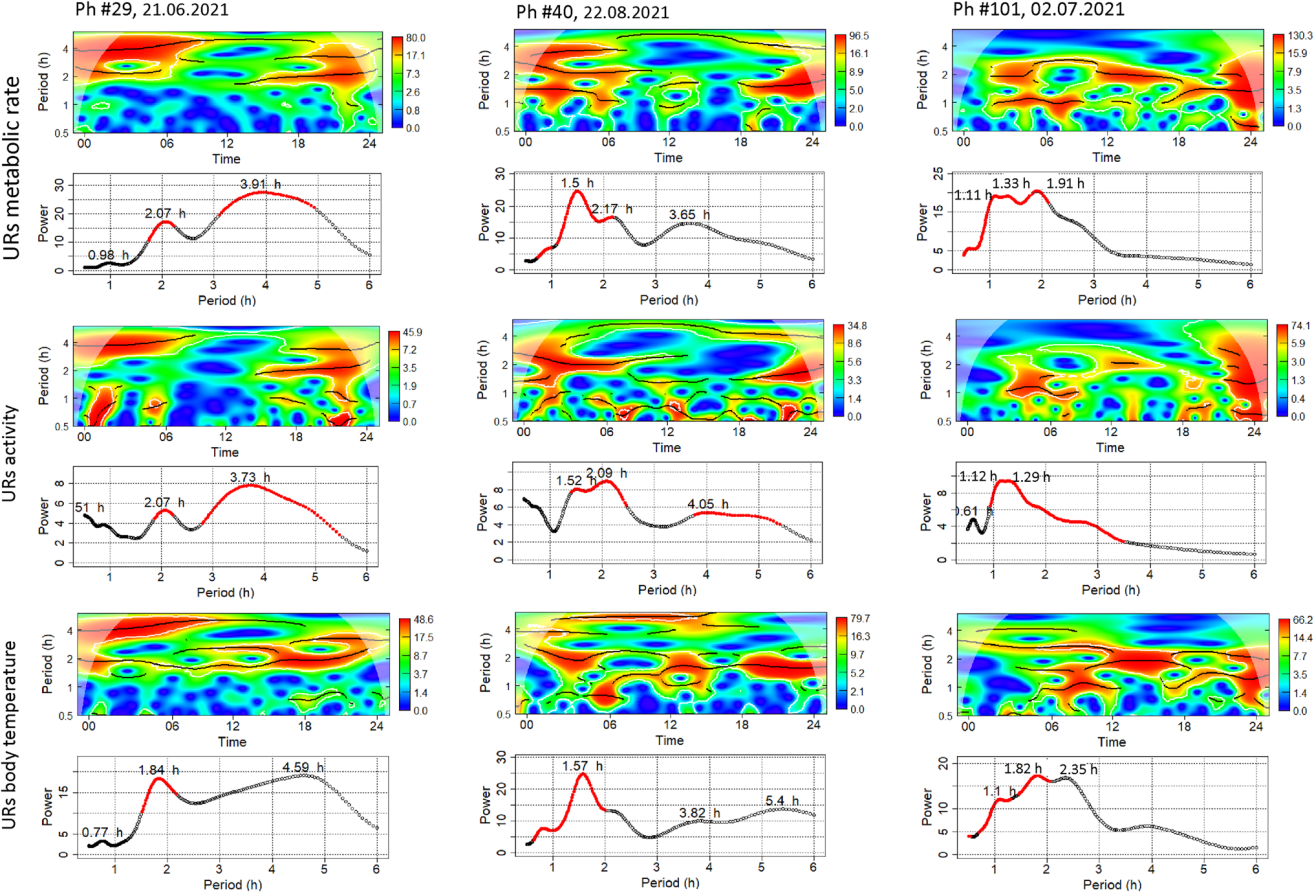
Wavelet analysis and power spectrum of ultradian periodicity of MR (top rows of graphs), locomotor activity (middle row of graphs) and T_b_ (bottom row of graphs) of the three 24 hourly records presented in Fig. 1. Each graph has two parts with a heatmap of the wavelet analysis on top and the periodogram at the bottom. Red areas in the heatmap indicate the presence of significant periodicity (p < 0.05). Red sections of the lines indicate significant (alpha = 0.01) periods as determined from random shuffles of original values of MR, activity and T_b_

Wavelet analysis of MR revealed three different periodicities which can be associated with three URs of different amplitude. The original records showed UR*small* with a period of about 1 h (0.98, 1.5, 1.1 h in the three examples in Fig. 2), UR*medium* had periods of about 2 h (2.07, 2.17, 1.33 h in Fig. 2), and UR*large* periods of about 3 h (3.91, 3.65, 1.91 in Fig. 2). These examples demonstrate the individual variability of period length of all three URs.

Wavelet analysis of the 24hourly records of T_b_ and activity revealed that they also contained three different ultradian periodicities although these were not apparent in the original records (Figs. 1 and 2) Their period lengths were comparable to the periods retrieved from wavelet analysis of MR URs, e.g. hamster Ph#29 (Fig. 2) had a UR*small* period of 0.98 h for MR, 0.51 h for activity and 0.77 for T_b_. The corresponding values for UR*large* were 3.91 h, 3.37 h and 4.59 h.

Within each individual the ultradian rhythms of MR, T_b_ and locomotor activity were similar but not identical, e.g. UR*small* of hamster Ph#29 had a period of 0.98 h for MR, 0.51 h for activity and 0.77 h for T_b_, Corresponding periods for UR*medium* were 2.07 for MR, 2.07 for activity and 1.84 h for T_b_. For UR*large* the periods for MR, activity and Tb varied internally from 3.91, 3.73, and 4.59 h. (Fig. 2). Wavelet analysis showed additionally that the period length of ultradian rhythms was not constant throughout the day but varied, as well as there were changes in amplitude with lowest values observed during the diurnal resting phase of the night active Djungarian hamsters (Fig. 1).

### Long term variability of ultradian rhythms

**Fig. 3:**
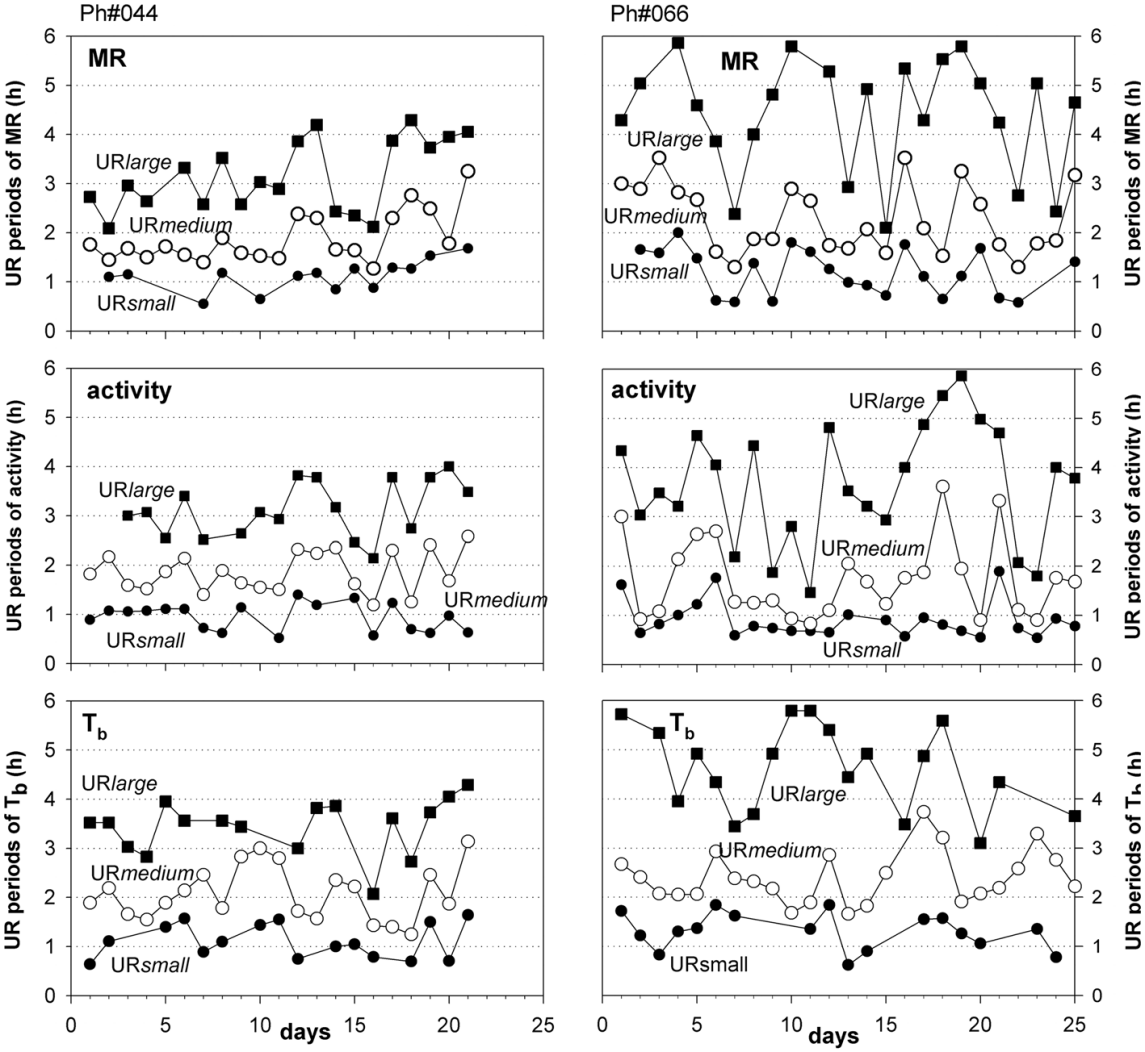
Development of UR period duration in two hamsters over the course of 21 days (Ph#44, male, mean M_b_ 31.5 g) and 24 days (Ph#66, female, mean M_b_ 33.6 g). The amplitude and frequency indicate three different ultradian rhythms for metabolic rate, locomotor activity as well as body temperature, filled square UR*large* ultradian large amplitude, unfilled circle UR*medium* ultradian medium amplitude, filled circle UR*small* ultradian small amplitude

The ultradian pattern of individual hamsters varied from day to day (Fig. 3), although the hamsters were kept in a constant environment with ad libitum food and water supply, suggesting an endogenous origin of these variations. Wavelet analysis was performed for each single 24hourly record revealed almost daily changes of ultradian periods for URs of MR, activity as well as T_b_. Many of these changes occurred in parallel in UR*small*, UR*medium* and UR*large*. The time course of UR periods revealed a pattern where groups of days with relatively short ultradian periods alternated with groups of days with long ultradian periods, suggesting infradian influences on ultradian periodicity.

**Fig. 4:**
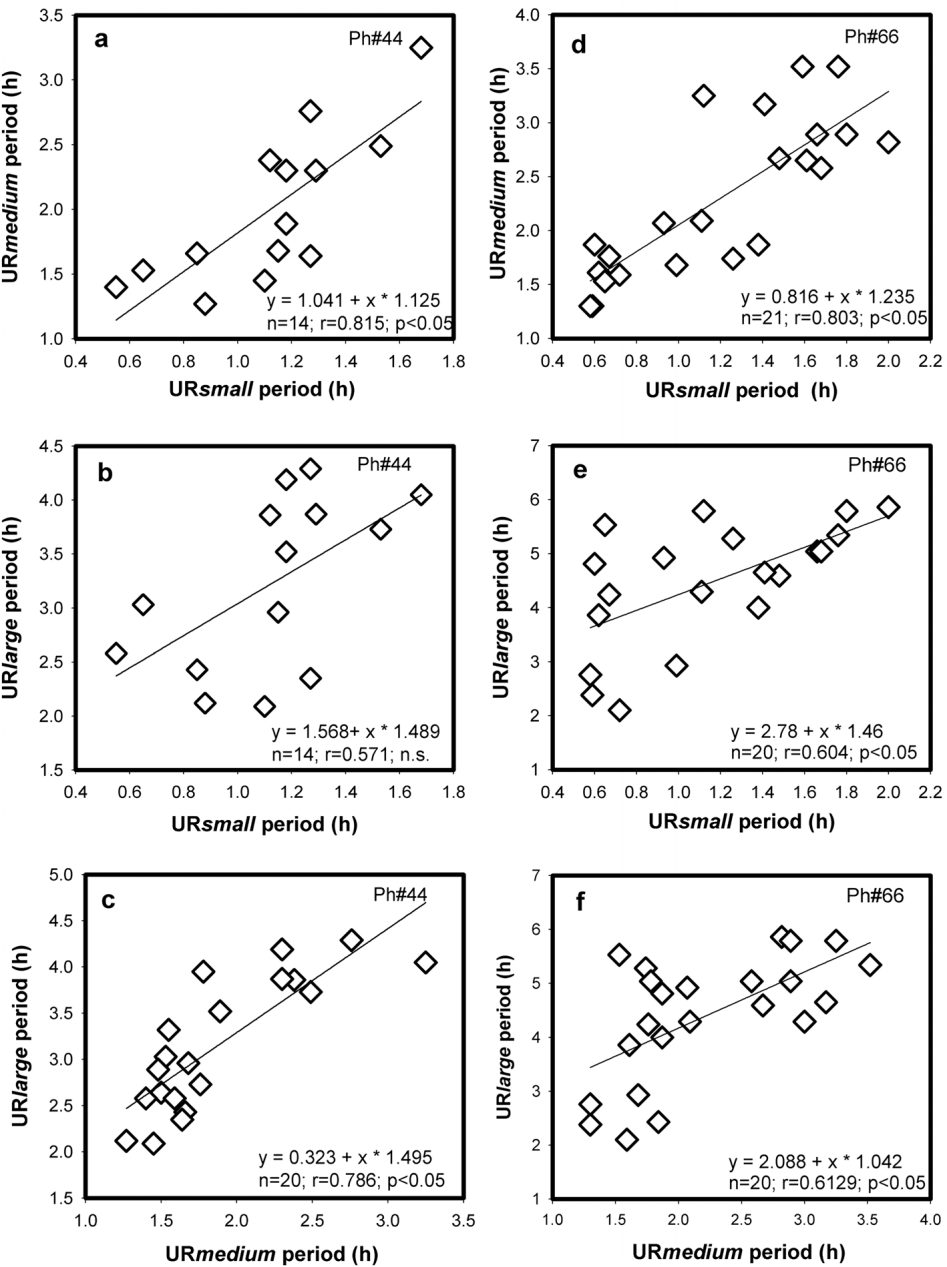
Correlations between the three ultradian periods UR*small* versus UR*large*, UR*small* versus UR*medium*, and UR*medium* versus UR*large* obtained from records of metabolic rate over the course of 21 days (Ph#44) and 25 days (Ph#66). Time course of data see Fig. 3

The apparent synchrony between UR*small*, UR*medium* and UR*large* was analysed by pairwise comparisons of the time course of UR periods. UR*small* versus UR*medium*, UR*small* versus UR*large*, and UR*medium* versus UR*large*, as shown in the six graphs from 2 hamsters in Fig. 4. If the three different UR categories would be harmonics of one ultradian oscillator one would expect straight linear regressions and correlations of periods between UR categories. The scattered data points do not support this view. In five of the six comparisons UR periods were significantly correlated, i.e. they are in synchrony with each other but the scattered data points doubt that they operate as fractions of each other. In one hamster the MR periods of UR*small* versus UR*large* were even lacking evidence for correlation.

This interaction between ultradian periods of MR, activity and T_b_ was evaluated in 8 hamsters. Individual 24hourly records were obtained from recordings which lasted between 14 and 24 days. 72 pairwise comparisons revealed 47 significant correlations (p < 0.05), i.e. the daily changes of UR periods of MR, activity and T_b_ over time were synchronized in 65.3% of all cases. Best synchronisation was found between UR*small*, UR*medium* and UR*large* of MR (p < 0.05 in 20 of 24 pairwise comparisons), whereas URs of activity were correlated only in 13 out of 24 cases, and the time course of UR periods for T_b_ was significant in 14 out of 24 pairwise comparisons (p < 0.05). This suggests the existence of more than one cellular or central oscillator for ultradian rhythms.

### Amplitude of ultradian rhythms

**Fig. 5:**
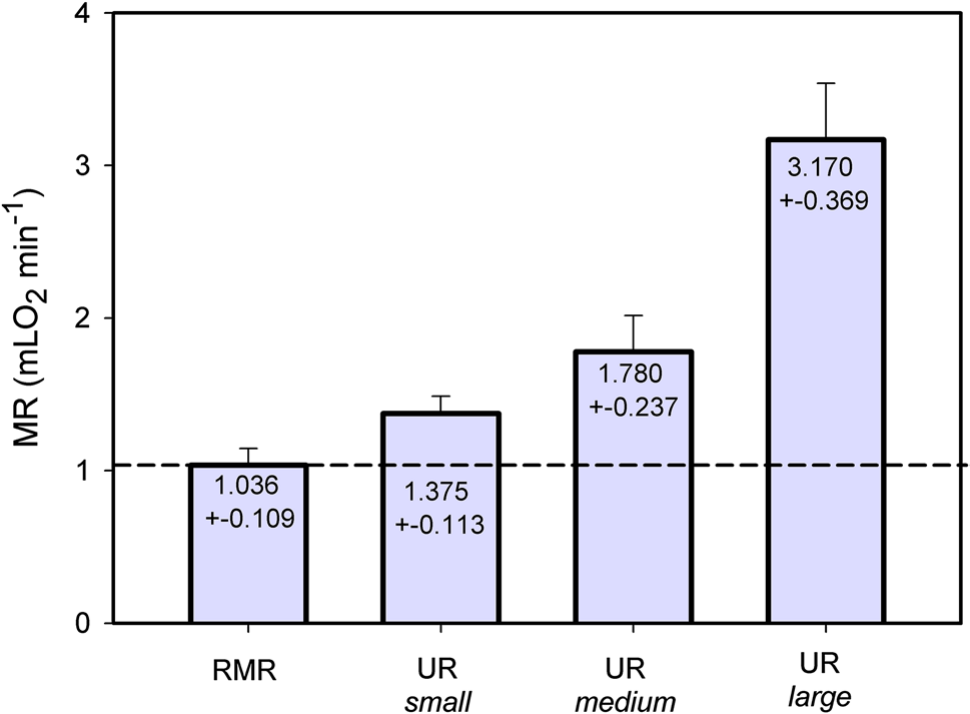
Maximum MR during URs. The level of RMR and the amplitude of UR*small*, UR*medium* and UR*large* were calculated on the basis of individual periodograms and wavelet analysis for at least 14 days. Mean values ± SD of 8 hamsters. Differences between RMR, UR*small*, UR*medium* and UR*large* were significant with p < 0.001 (n = 8; one way ANOVA for repeated measures, pairwise comparisons by Holm-Sidak method)

The results of wavelet analysis were used to determine the amplitude of ultradian bursts and to analyse further properties which differentiate between UR*small*, UR*medium* and UR*large* (Table 1). The amplitude of URs was determined by searching the peak MRs in each 24hourly record with search windows adapted to the periods of UR*small*, UR*medium* and UR*large* for this particular 24 h record. Following peak identification a similar procedure was used to identify the minimum MR prior to the peak, which allowed to calculate the amplitude of changes in MR, activity and T_b_ during ultradian bursts (Table 1 and Fig. 5). The three UR categories showed peak values of MR, T_b_ and activity with three different magnitudes as well as three different amplitudes of change. This supported the initial discrimination of ultradian bursts into the categories small, medium and large.

**Table 1:** Properties of UR*small*, UR*medium* and UR*large* of metabolic rate (MR), and interaction with URs of T_b_ and locomotor activity. URs were determined in 24hourly records of n = 8 hamsters by wavelet analysis. The individual period lengths of these URs were used to identify single ultradian bursts, their minimum and peak for MR, Tb and activity. For statistical analysis one way ANOVA repeated measures for three groups and pairwise multiple comparison with the Holm-Sidak method were used. Means ± SEM. Results from activity amplitude failed normal distribution and therefore the Tukey method was used

	UR*small*	UR*medium*	UR*large*	
n hamsters, N bursts	n = 8, N = 79	n = 8, N = 57	n = 8, N = 64	
Ultradian bursts per day	> 9.8 ± 3.6*	> 7.1 ± 1.8*	8.0 ± 1.9	
Period duration [min]	74.9 ± 9.11	132.9 ± 14.2	203.2 ± 22.2	*p* < *0.01*
Peak MR [mL O_2_ min^−1^]	1.375 ± 0.039	1.780 ± 0.084	3.170 ± 0.131	*p* < *0.01*
MR amplitude [mL O_2_ min^−1^]	0.231 ± 0.023	0.520 ± 0.033	1.915 ± 0.147	*p* < *0.05*
T_b_ amplitude [°C]	– 0.04 ± 0.05	0.29 ± 0.06	1.12 ± 0.14	*p* < *0.05*
Activity amplitude [I min^−1^]	1.19 ± 0.42	0.301 ± 0.06	15.47 ± 1.08	Small vs medium = *n.s.*, other pairs *p* < *0.05*

*Only bursts close to baseline level were considered. The true number of UR*small* and UR*medium* may be greater due of overlap with UR*large*

The original records of MR, activity and T_b_ (Figs. 1, 2) suggest a lower amplitude or lower intensity of URs during daytime hours (08:00…16:00) i.e. the circadian resting phase of Djungarian hamsters. To quantify this diurnal regression URlarge periods (Fig. 1) were separated into daytime values and nighttime values (Table 2). The peak MR, as well as the amplitude of MR, T_b_ and activity were significantly reduced during daytime hours suggesting an attenuation of UR*large* during the resting phase by about 40%.

**Table 2:** Ultradian bursts during the circadian activity phase versus the resting phase. The results are from the same hamsters as described in Table 1. Hamsters are nocturnal. The light phase from 08:00….16:00 was considered as their resting phase. UR*large* bursts occurring during the resting phase were compared with UR*large* bursts during the activity phase (00:00…..07:59 and 16:01…..24:00). Amplitudes are the difference between minimum and maximum MR, T_b_ and activity of each burst. Mean ± SEM. Paired t-test. Results for the T_b_ amplitude were not normally distributed and therefore compared using a Wilcoxon test

	Resting phase	Activity phase	Significance	Reduction during resting phase
n hamsters, N UR*large* bursts	n = 8, N = 22	n = 8, N = 42		
peak MR]mLO_2_ min^−1^]	2.31 ± 0.17	3.24 ± 0.17	*p* = *0.002*	28.7%
MR amplitude [mLO_2_ min^−1^]	1.35 ± 0.18	2.18 ± 0.16	*p* = *0.003*	38.1%
T_b_ amplitude [℃]	0.80 ± 0.10	1.37 ± 0.16	*p* = *0.008*	0.57 °C
Activity amplitude [I min^−1^]	7.51 ± 1.42	18.74 ± 1.41	*p* = *0.002*	59.5%

**Fig. 6:**
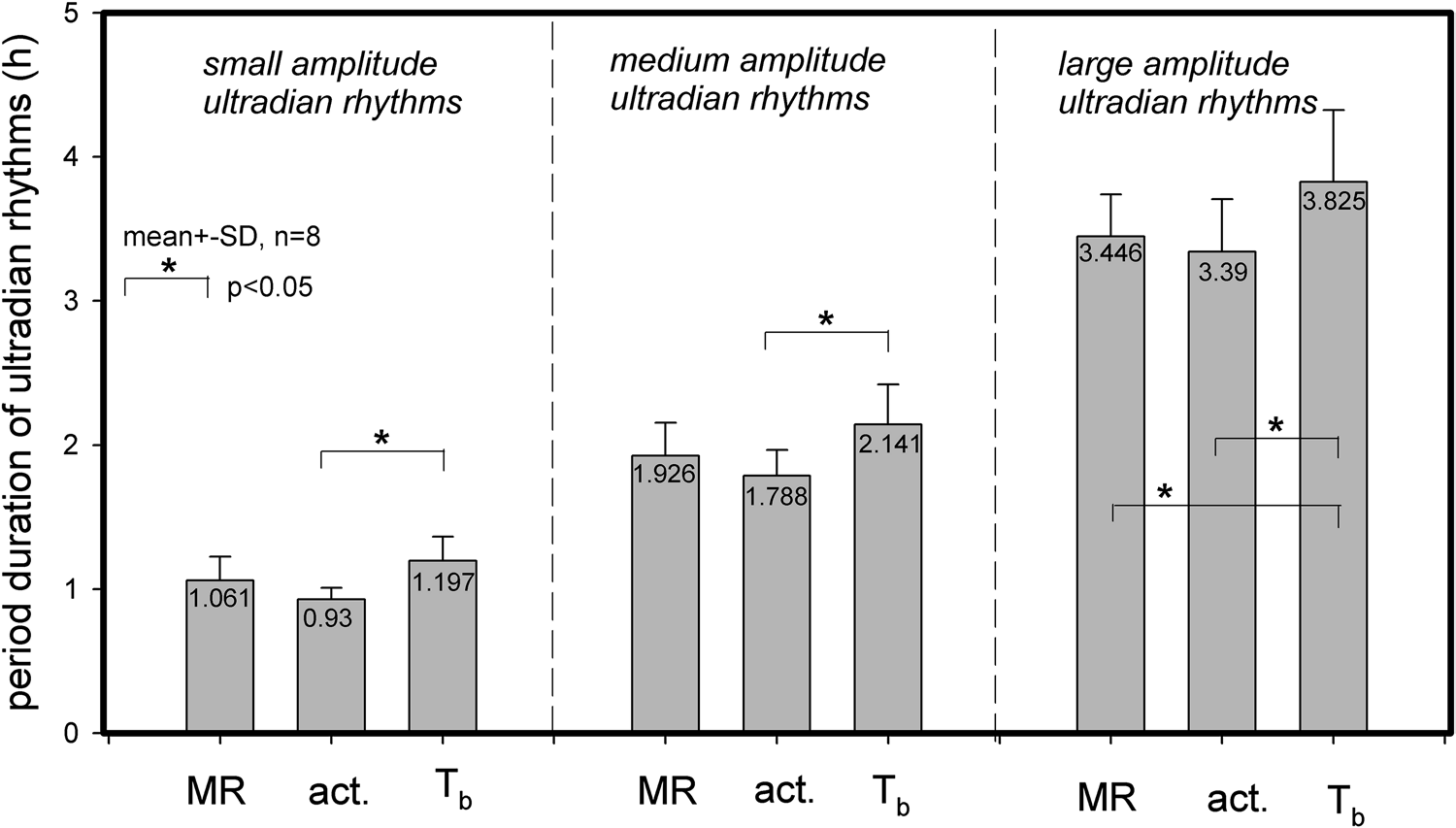
Mean values ± SD of ultradian period durations for MR (VO_2_), locomotor activity (act) and body temperature (T_b_) from 8 hamsters. SD represents the variation between individuals. Data were obtained from similar evaluations as presented for two hamsters in Fig. 3. Data with in UR*small*, UR*medium* and UR*large* were compared by repeated measures ANOVA. Differences between group specific periods were analysed by the Holm-Sidak Method and significant (p < 0.05) differences were marked by connecting bars

The time course of UR periods (Fig. 3) suggests that calculation of mean values for UR periods, as well as the statistical discrimination between URs of MR, and activity and T_b,_ requires prolonged recordings to compensate for infradian influences on ultradian periods. Therefore, individual means for the period duration of UR*small*, UR*medium* and UR*large* were calculated for MR, activity and T_b_ from 8 hamsters recorded for 14 and 24 days without interruption This calculation revealed significant differences between UR*small*, UR*medium* and UR*large* for MR, T_b_ and activity (Fig. 6). When comparing the corresponding UR periods within UR*small*, UR*medium* and UR*large* the periods for T_b_ were significantly longer than the corresponding UR periods of activity in all three UR categories. In UR*large* the period for T_b_ was also longer than the corresponding MR period. At present it is not clear if this indicates separate ultradian oscillators or if it is a side action of thermal inertia in contrast to the immediacy of activity and metabolic responses.

### Dynamics of ultradian bursts

**Fig. 7:**
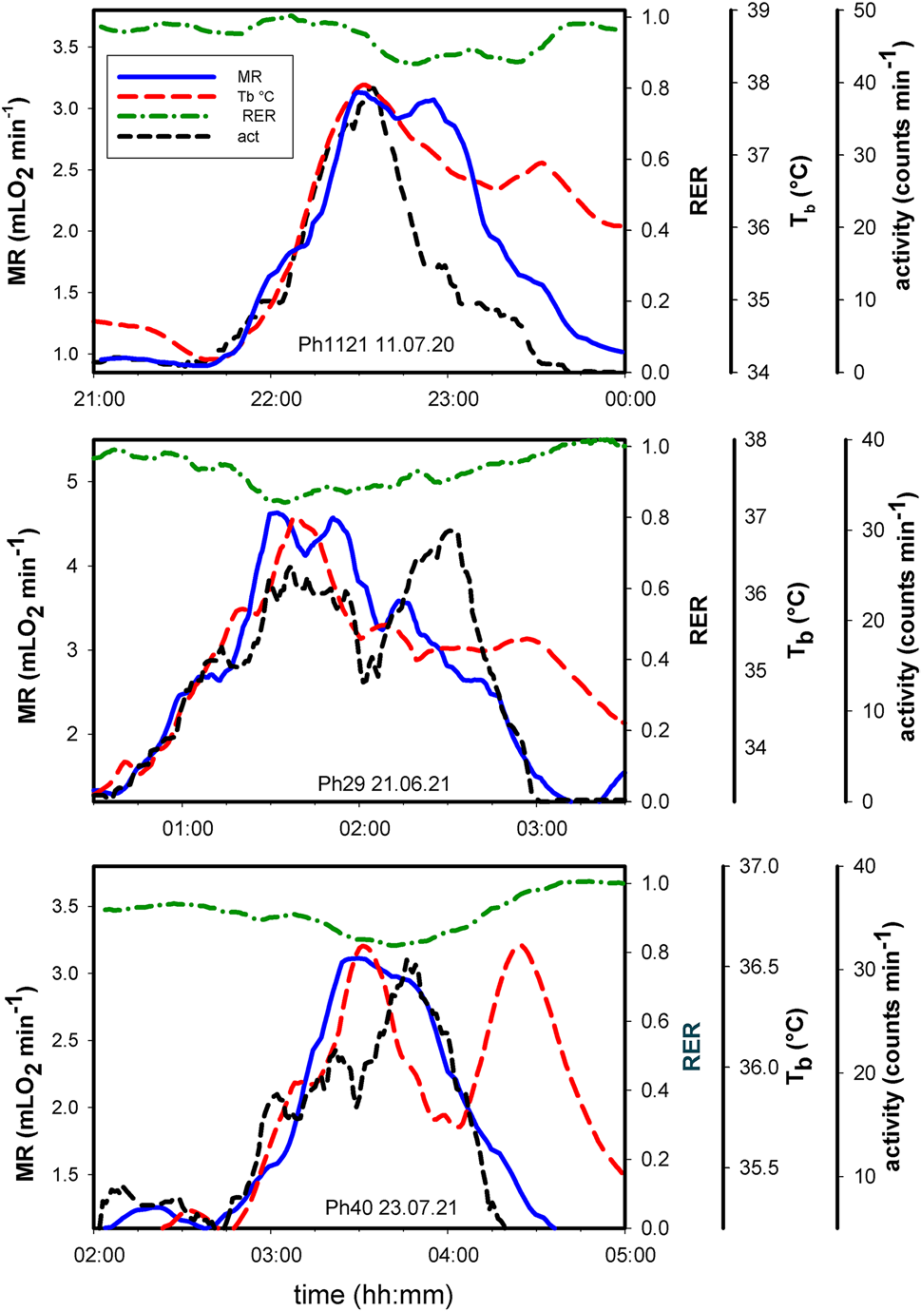
UR*large* amplitude dynamics. Three examples of changes in metabolic rate, RER, body temperature and locomotor activity during ultradian large amplitude bursts. Data for MR, Tb and act were scaled to similar amplitude to facilitate the comparison of changes during individual ultradian bursts

URs of large amplitude (UR*large*) were starting with a simultaneous increase of locomotor activity, metabolic rate and body temperature, and the RER was gradually decreasing, as shown for three different examples in Fig. 7. The large amplitude bursts lasted about 1–2 h and each parameter returned to its initial level. The return differed between the three physiological parameters, especially T_b_ remained elevated for an extended period of time.

**Fig. 8:**
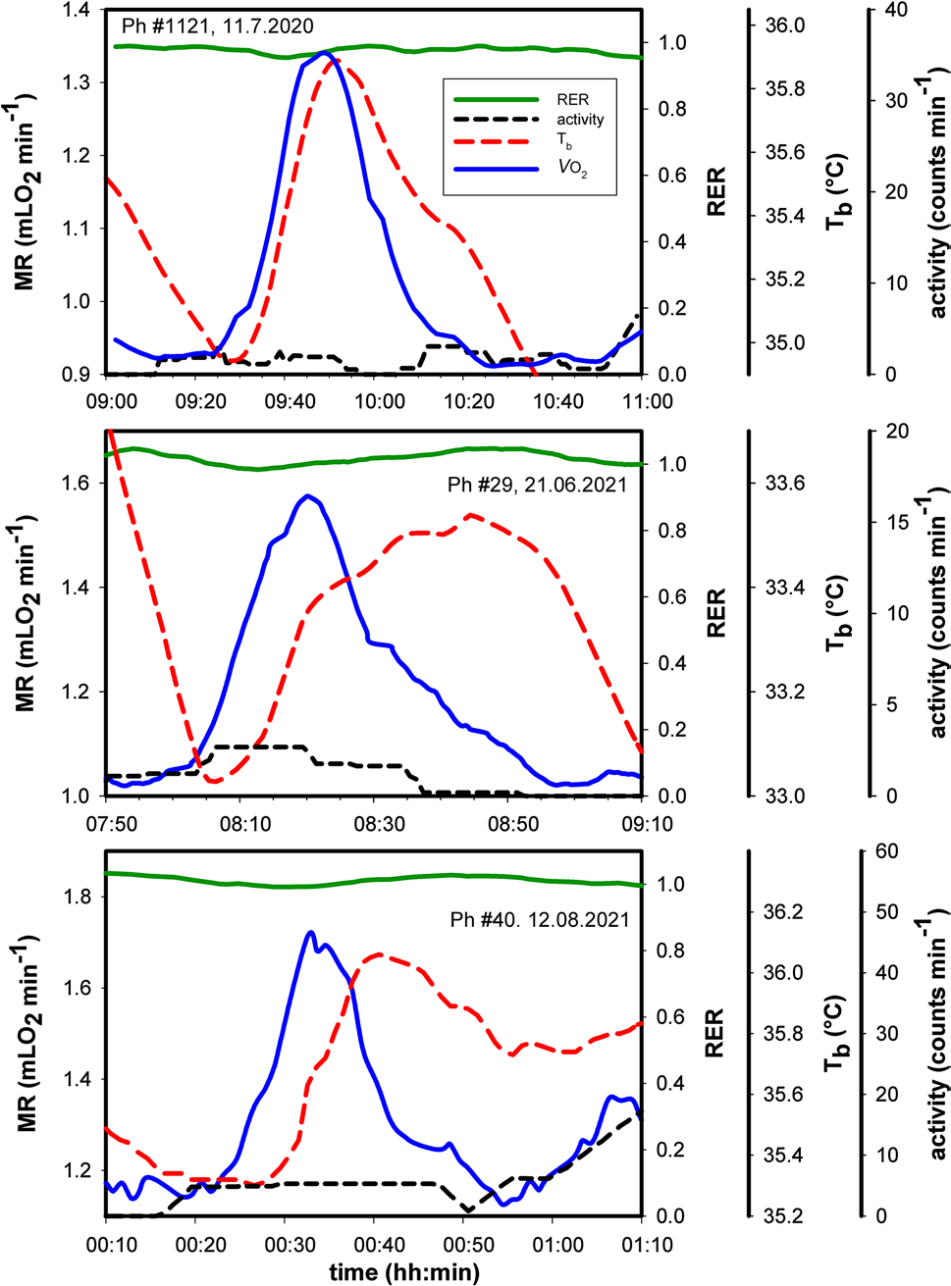
UR*small* amplitude dynamics. Three examples of changes in metabolic rate, RER, body temperature and locomotor activity during ultradian small amplitude bursts. Further details see legend of Fig. 8

URs of small (UR*small*) and medium (UR*medium*) amplitude frequently showed a different pattern, with a predominant initial increase of metabolic rate, and lacking or delayed changes in locomotor activity and T_b_ (Fig. 8). During 68% of UR*small* metabolic bursts (79 bursts from 8 hamsters) the increase of MR was not accompanied by an increase of T_b_ and 44% lacked an increase in locomotor activity, although MR was elevated by about 20%, during an initial rise time of MR from minimum to peak MR which lasted 13.12 ± 3.60 min. This explains the negative mean values of T_b_ amplitude and the minor changes of activity for UR*small* in Table 1. Metabolic bursts of UR*medium* were lacking an increase of locomotor activity in 21% of 57 cases analysed, whereas in UR*large* the increase of MR was always accompanied by a simultaneous increase in locomotor activity and T_b_ as shown in Fig. 8.

**Fig. 9:**
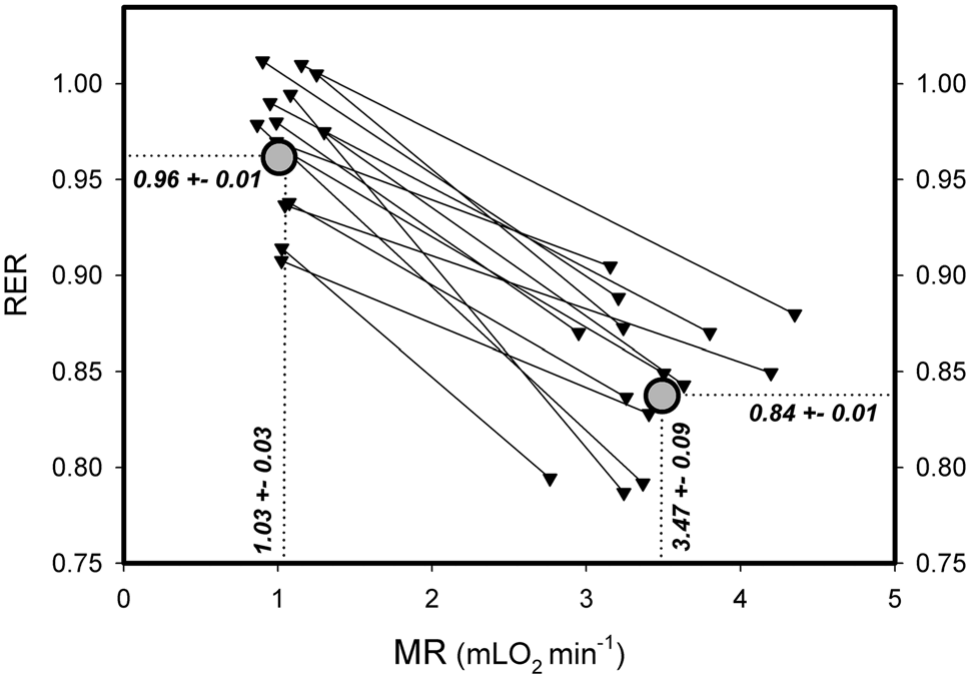
RER during large amplitude bursts of metabolic rate. MR and RER before an ultradian burst are compared with maximum metabolic rate and the associated minimum RER. 14 large amplitude variations were analysed from 8 different hamsters. Mean values were calculated for 8 hamsters

Large amplitude bursts were also characterized by a decrease of RER as summarized in Fig. 9, indicating enhanced lipid oxidation during UR*large*. An increase of mean MR from 1.03 to 3.47 mLO_2_ min^−1^ during UR*large*, was paralleled by a reduction of RER from 0.96 to 0.84. The hamsters were fed ad libitum with pellet food (breeding diet with high protein content) and burned primarily carbohydrates at the resting level of MR as indicated by the RER of 0.96. The reduction of the RER to 0.84 during the 3.38fold increase of MR indicates a major rerouting of metabolism to combustion of lipids. This occurred without any external stimulation indicating that metabolic fuel processing spontaneously altered between carbohydrate and lipid metabolism due to the activity of URs. In few cases we observed no reduction of RER during UR*large* which might have been caused by simultaneous food uptake, absorption and metabolization of carbohydrates in the pellet diet.

### Ultradian activity pattern and energy balance

The total amount of energy expenses for ultradian variations of MR was estimated from the difference between total energy expenses per day (DEE) and the energy expenses for RMR. The DEE of hamsters at 15 °C Ta was 44.4 ± 4.0 kJ day^−1^ and their RMR was 29.8 ± 2.7 kJ day^−1^ (n = 8, M_b_ 31.7 ± 2.5 g). This means that the ultradian system required 14.6 ± 3.1 kJ day^−1^, i.e. 32.7% of the daily energy budget at a T_a_ of 15 °C (calculations are based on the data used for Fig. 5).

**Fig. 10:**
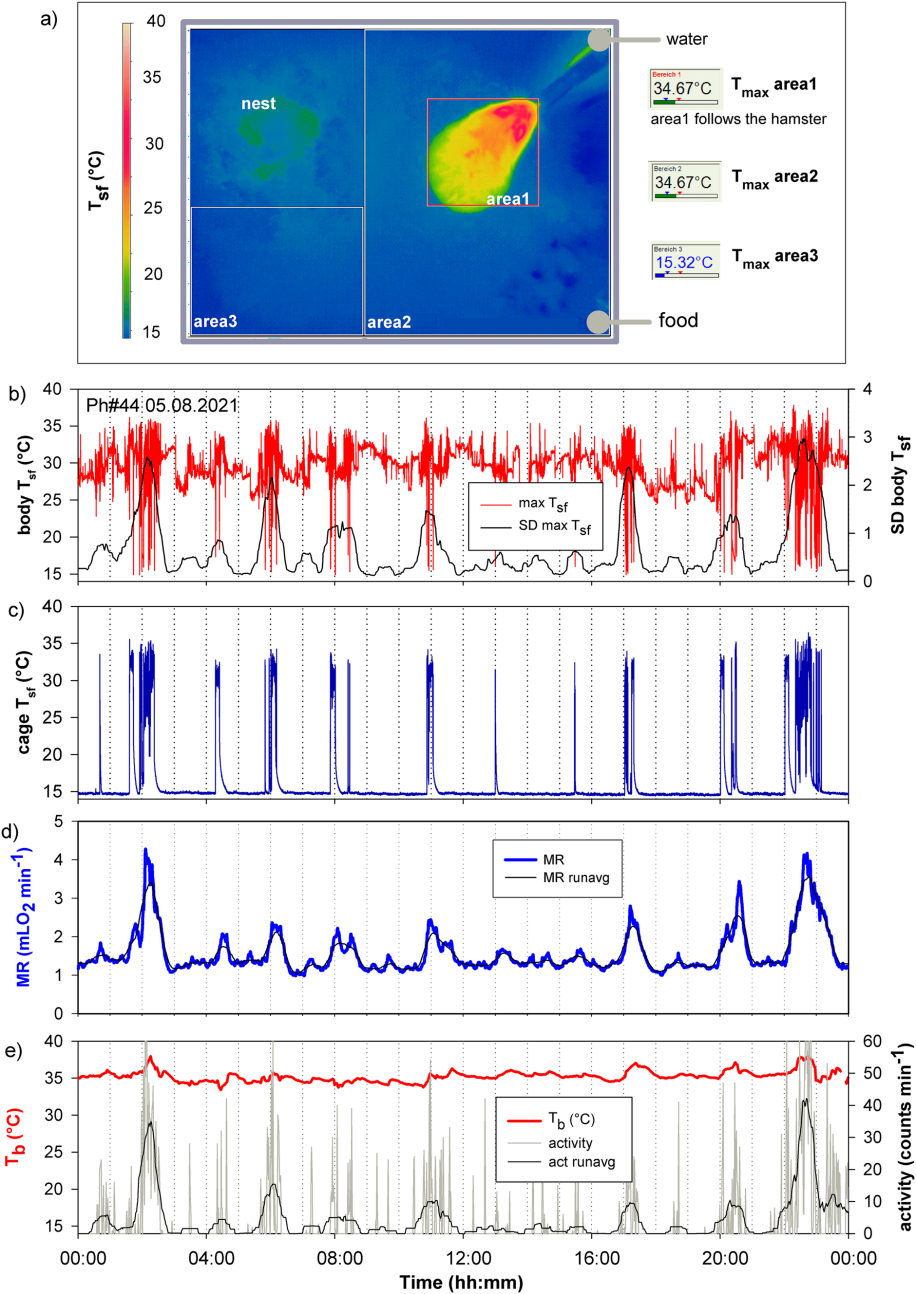
Activity behaviour of a hamster (Ph#44, male, Mb 28.6 g) recorded with an infrared thermovision camera (setup see Fig. 10a), Pictures were scanned and stored in one second intervals. Three areas of interest were defined. Area1 was programmed to follow the hamster, area2 and area3 were fixed and covered the cage bottom outside the nest. Maximum T_sf_ was read every second from the hamster (Fig. 10b, red line) and the cage bottom. T_sf_ of eyes and face of the hamster are close to T_b_ recorded with implanted transmitters (Fig. 10b). Most of the time hamster T_sf_ was below T_b_ (Fig. 10e) because only the fur surface was in sight of the thermovision camera. SD for body T_sf_ was calculated as an index for activity (Fig. 10b black line). Cage T_sf_ showed stable values at ~ 15 ℃, interrupted by rises above 30 ℃ which happened when hamster was active outside the nest (Fig. 10c). MR of the hamster (Fig. 10d). The blue line are readings per min and the black line is average MR (30 min running average) derived from the readings per min. Figure 10 e: core T_b_ of the hamster (red line) and locomotor activity both measured with transmitter telemetry. Activity is presented with two lines. Activity counts per min (grey line) and 30 min running average of activity counts per min (black line)

Activity of hamsters was recorded with implanted transmitters. They responded to any movement of the body and may not have mirrored the energy required for any kind of activity. To obtain further details about activity we additionally recorded the position and movements of hamsters with infrared thermovision which allowed to trace the posture and position of the hamster in the cage and allowed to discriminate if hamsters were active inside or outside their nest. Both methods were applied simultaneously to measurements of MR and T_b_ (Fig. 10). Telemetry and MR was recorded in 1 min intervals, IR images were recorded in 1 s intervals. To automatize the evaluation of IR images we defined three areas, i.e. one area which traced the hamster and 2 stable areas representing the cage area outside the nest. Maximum body T_sf_ of the hamster varied between 35.5 °C, i.e. close to core T_b_, and 15 °C close to T_sf_ of the cage bottom, depending upon the posture towards the thermovision camera (area 1 in Fig. 7a, and red line in Fig. 7b). Activity was calculated from the amplitude of T_sf_ changes (Fig. 7b black line), i.e. the standard deviation of T_sf_ min^−1^ This estimate of activity closely parallels the activity pattern obtained with implanted transmitter (Fig. 7e) and the ultradian pattern of MR (Fig. 7d).

The 7 UR*large* bursts of this hamster during 24 h were always associated with activity outside the nest. During the diurnal resting phase the hamster was less active outside the nest. In total it spent 195 min outside the nest for feeding, drinking, defecation and other activities (mean 177 ± 25 min in four hamsters). Thus, the hamsters remained in the nest for ~ 21 h per day, where they were active too with small movements, grooming and nest building. A motionless rest in the nest was rarely seen and limited to short periods of a few seconds or minutes. This can be concluded from the amplitude of hamster T_sf_ which was > 1 °C most of the time. In a motionless hamster one would expect T_sf_ changes < 0.5 °C sec^−1^, similar to stable readings of cage bottom T_sf_ which varied < 0.5 °C sec^−1^.

**Fig. 11:**
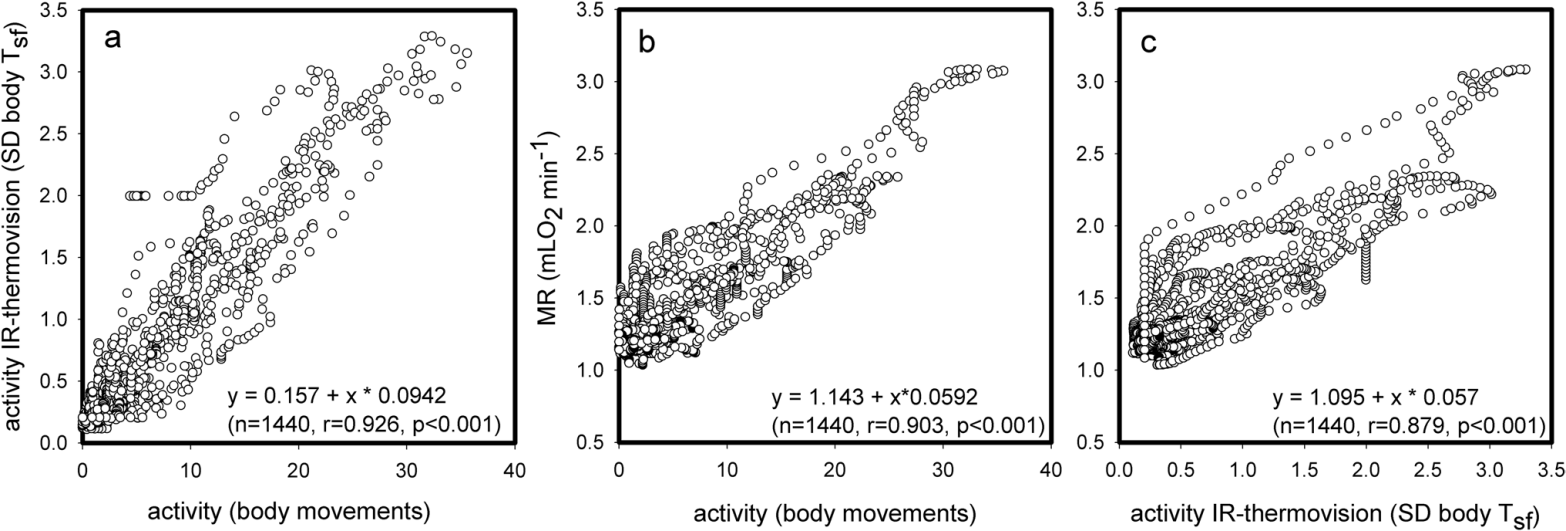
Comparison of locomotor activity measured by transmitter and infrared thermovision (Fig. 10a). Relation between MR and activity either measured with implanted transmitter (Fig. 10e) or infrared thermovision (Fig. 10b)

Activity records with implanted transmitters and activity records with the thermovision camera were closely correlated (Fig. 11a). Both methods had similar slopes for the relation between an increase in activity and an increase of energy requirements(Fig. 11 b, c), underlining the close relation between locomotor activity and gross ultradian changes of MR. The comparison of both methods revealed that infrared thermovision is a suitable method for the measurement of total activity in small rodents without requiring surgery, and provides additional information on location and behaviour of an animal its T_sf_ as well as core temperature.

### Response of URs to energetic challenge

**Fig. 12:**
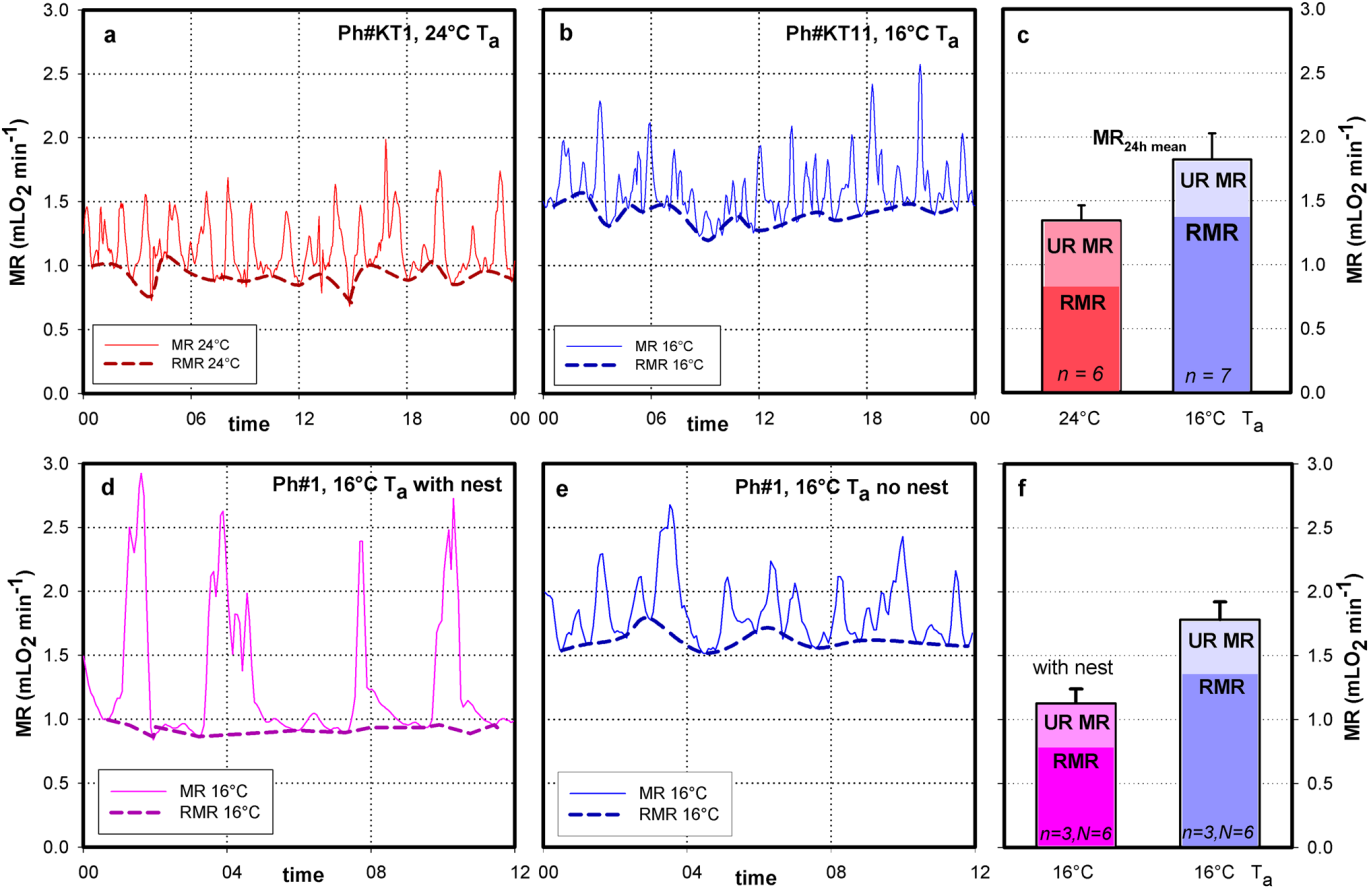
Effect of cold and nest building on hamster energy requirements. Panels a, b show 24 h records of MR of two hamsters at 24 ℃ (pink) or 16 ℃ T_a_ (blue), including RMR calculated from minimum MR (6 min averages). Graph c mean values for MR, RMR and UR MR. See also Table 1. Graph c, d 12 h records of one hamster at 16 ℃ Ta with nest (pink) and 16 ℃ without nest. Graph f mean values from 3 hamsters and 6 recordings. See also Table 2

Cold exposure will increase energy expenditure to maintain euthermia. It is unknown if this changes the periodicity or amplitude of ultradian metabolic processes. Short day acclimated hamsters were kept at 24 ℃ T_a_ (thermoneutral) or at 16 ℃ T_a_ (moderate cold exposure) in type 1 cages with a small amount of wood shavings as bedding material. MR and T_b_ was recorded continuously. The hamsters displayed ultradian variations of MR (see individual examples in Fig. 12). The RMR of hamsters was tracked by the minima of ultradian metabolic bursts, and a 24hourly mean of RMR was calculated. This was subtracted from the total metabolic rate per day (daily energy expenditure, DEE) to obtain the contribution of ultradian metabolic bursts. Moderate cold exposure by changing Ta from 24 to 16 ℃ increased DEE of hamsters from 34.8 kJ day^−1^ to 58.1 kJ day^−1^, as expected (Table 3, Fig. 12a, b, c). This was largely due to an increase of RMR from 0.845 mLO_2_ min^−1^ to 1.432 mLO_2_ min^−1^.

**Table 3:** Energy expenditure and metabolic URs of Djungarian hamsters at 24 ℃ T_a_ (thermoneutral) and 7 hamsters at 16 ℃ T_a_ (moderate cold). T_a_ was measured inside the cages. The differences between the two groups were compared by t-tests

Ambient temperature (T_a_)	24 °C (n = 6)	16 °C (n = 7)	
DEE [kJ d^−1^]	34.8 ± 13.9	58.1 ± 6.5	*p* = *0.001*
HP_24h mean_ [mW]	403.2 ± 140.5	671.4 ± 75.7	*p* = *0.001*
MR_24h mean_ [mLO_2_ min^−1^]	1.345 ± 0.118	1.822 ± 0.204	*p* < *0.001*
RMR_24h mean_ [mLO_2_ min^−1^]	0.845 ± 0.126	1.423 ± 0.249	*p* = *0.001*
Ultradian MR_24h mean_ [mLO_2_ min^−1^]	0. 511 ± 0.120	0.409 ± 0.129	*n.s*
Ultradian MR as % of MR_24h mean_	37.9%	22.4%	
RER	0.928 ± 0.029	0.893 ± 0.034	*n.s*
Mean peak MR [mLO_2_ min^−1^]	1.625 ± 0.149	2.129 ± 0.226	*p* < *0.001*
Number of peaks [n]	33.5 ± 1.4	31.3 ± 6.7	*n.s*
Period UR*small* [h]	0.803 ± 0.128	0.837 ± 0.208	*n.s*
Period UR*medium* [h]	1.345 ± 0.217	1.507 ± 0.515	*n.s*
Period UR*large* [h]	2.312 ± 0.458	2.673 ± 0.971	*n.s*

The energy requirements of ultradian metabolic variations were 0.511 and 0.409 mLO_2_ min^−1^ at 24 °C and 16 °C Ta, respectively (difference not significant). Thus, surprisingly the energy expenditure of URs remained constant at both temperatures. It was only the rise of RMR which could be held responsible for the increase of total energy requirements in moderate cold. Due to the rise in RMR the relative amount of ultradian energy expenses decreased from 37.9% of DEE at thermoneutrality to 22.4% of DEE in moderate cold (Table 3). The periods of UR*small*, UR*medium* and UR*large* as well as their pattern was similar in both groups (Table 3, Fig. 12a, b and c).

URs are tightly linked with behavioural activity of animals. We stimulated behavioural activity of hamsters at 16 ℃ by offering them nesting material (cellulose bulk). The presence of a nest reduced MR but increased the incidence of spontaneous daily torpor in hamsters. Therefore, we compared only 12 h records during the nocturnal euthermic phase (Table 4, Fig. 12d, e, f). Three of the hamsters included in the previous group at 16 ℃ T_a_ received nesting material and immediately built a well insulating nest which reduced total MR from 1.822 to 1.092 mLO_2_ min^−1^. The period of URs as well as the metabolic expenses of UR MR remained unchanged, similar to the temperature effect described in Table 3.

**Table 4:** Metabolic rate and metabolic URs of Djungarian hamsters at 16 ℃ either without or with a nest. Results from 12 h records in three hamsters, each hamster was recorded twice (n = 3, N = 6). Mean body mass was 28.4 g. Means ± SD

	T_a_ 16 °C (n = 3, N = 6) no nest	T_a_ 16 °C with nest
T_b 12 h mean_	35.9 ± 0.3	35.6 ± 0.1
MR_12h mean_ [mLO_2_ min^−1^]	1.782 ± 0.204*	1.092 ± 0.140*
RMR_12h mean_ [mLO_2_ min^−1^]	1.415 ± 0.207*	0.829 ± 0.091*
Ultradian MR_12h mean_ [mLO_2_ min^−1^]	0.297 ± 0.080	0.263 ± 0.068
Ultradian MR as % of MR_12h mean_	16.0%	24.1%
RER_12h mean_	0.947 ± 0.018	0.946 ± 0.018
Average peak MR [mLO_2_ min^−1^]	2.124 ± 0.301*	1.624 ± 0.072*
Number of peaks per 12 h > 2 mLO_2_ min^−1^	10.25 ± 3.63*	4.00 ± 1.77*
Period UR*small* [h]	0.772 ± 0.245	0.892 ± 0.099
Period UR*medium* [h]	1.312 ± 0.433	1.508 ± 0.054
Period UR*large* period [h]	2.418 ± 0.694	2.439 ± 0.006

*Values differ significantly in hamsters kept with and without nest p < 0.05

However, the pattern of URs changed with the presence of a nest. URs were dominated by a few large peaks of MR (Fig. 12). This reduced the average peak MR from 2.124 mLO_2_ min^−1^ to 1.624 mLO_2_ min^−1^ whereby the total amount of MR UR remained unchanged (Table 2). The visual impression of a different pattern is underlined by counting the number of ultradian MR peaks > 2.0 mLO_2_ min^−1^. This arbitrary threshold revealed 4 peaks per 12 h in hamsters using a nest instead of 10 peaks per 12 h in hamsters living without a nest.

## Discussion

### Multiple ultradian rhythms

Djungarian hamsters showed similar URs in metabolic rate (MR), locomotor activity and T_b_. MR revealed the most detailed insights into the complexity of ultradian organisation of body functions. At least three different URs were running in parallel with different amplitudes and variable frequencies. They could be differentiated by amplitude and period. Rhythms of small (UR*small*), medium (UR*medium*) and large (UR*large*) amplitude were associated with short (~ 1 h), medium (~ 1.5–2.2 h) and large (~ 2.0–5.0 h) periods, respectively. These were running in parallel in MR, T_b_, as well as activity. This differs from previous studies in Djungarian hamsters and other small mammals where always one systemic UR was described with a period ranging between 1 and 6 h (Prendergast and Zucker 2012, Gerkema et al. 1990b, Honma and Hiroshige 1978).

The metabolic ultradian system required a substantial amount of the total daily energy budget. Most hamsters required a constant amount of 14.6 kJ day^−1^ for their MR URs, i.e. up to 30% of the total daily energy requirements. Moderate cold exposure as well as the lack of a nest enhanced the MR for heat production to maintain euthermia. This was solely achieved by an increase of RMR, whereas ultradian MR remained unchanged at the thermal challenges presently used. Previous studies in Djungarian hamsters have shown that the difference between DEE and RMR increases in severe cold (− 1 ℃ through – 15 ℃ T_a_) suggesting an even greater cost of activity during URs (Ruf and Grafl 2010).

The discovery of different URs may partly be explained by technical reasons and the high resolution of our recording device for MR. We recorded MR, T_b_ and activity at 30 s or 1 min sampling intervals continuously over several months. Previous long-term studies on ultradian rhythms used longer sampling intervals for food intake, wheel running or locomotor activity by passive IR detectors which required whole body displacement (Gerkema et al. 1990a, Gerkema et al. 1991, Prendergast and Zucker 2012, Ono et al. 2015). URs of endocrines depend upon sampling of body fluids which are only accessible at larger intervals of 10, 20 or 30 min., which can only be continued for a limited period of time. This limits the resolution for differentiation of URs and focusses interpretation on one unique UR. Our study further shows that wavelet analysis including a power spectrum provides a more detailed insight into the dynamics of URs than Fourier analysis, Lomb-Scargle periodograms or other methods of time series analysis.

URs measured on the cellular level or in biochemical pathways can be shorter than systemic URs. Protein biosynthesis in dense cultures of rat hepatocytes cycle with periods ranging between 30 min and 1 h (Brodsky 2014). Similar URs were found in the dry weight of acinar cells of the parotid gland in rats or in UV-absorption of crawfish mechanoreceptor neurons (Brodsky 2014). Similar short period URs were observed in blood levels of insulin and catecholamines (Simon and Brandenberger 2002; Schöfl et al 1997).

The day-by-day variation of URs occurred in all three URs to a similar extent (Fig. 3). The variability of their correlation suggests that there are at least three autonomous UR oscillators which are mutually synchronized or switched on and off by a network of interactions, instead of being a fraction or harmonic of one single UR (Fig. 4). The existence of an ultradian master clock, similar to the SCN for circadian rhythms, is unlikely, at least at the present state of knowledge. However, there is evidence for the coordination of URs on the cellular and tissue level (Isomura and Kageyama 2014; Yang et al. 2022) as well an interaction of several brain areas and endocrine networks (Grant et al. 2018; Goh et al. 2019) which could be held responsible for URs with different periods. These activities may be superimposed by the dopamine ultradian oscillator (DUO) which controls arousal, sleep and activity in a period range of ~ 4 h (Blum et al. 2014; Prendergast and Zucker 2016). This periodicity corresponds with the UR*large* in our present study.

### Neural control of ultradian rhythms

Our present findings indicate that several URs are oscillating in an animal at the same time, in cells as well as on the systemic level. This raises the question if they are running in parallel, independent from each other, or if they are linked by endocrine or neural control. The 24hourly records of MR, T_b_ and activity records in Fig. 1 show high amplitude URs during the nocturnal activity phase and low amplitudes during the diurnal resting phase, indicating an interaction between the circadian and the ultradian system.

Ablation of the circadian pacemaker in the SCN eliminates CRs but URs of activity behaviour and feeding persisted in 8 out of 11 SCN-ablated voles (Gerkema et al. 1990b, Gerkema and van der Leest 1991). Similar observations were made in golden hamsters and rats (Refinetti et al 1994; Stephan and Zucker 1972). In contrast SCN ablation in LEW/Ztm rats abolished circadian as well as ultradian components of activity rhythms (Wollnik and Turek 1989). The discrepancy may be due to the extent of surgical ablation in the hypothalamus. Detailed histological analyses of ablation studies in voles (Gerkema et al. 1990b) showed that hypothalamic regions outside the SCN were generating URs which exerted ultradian effects on the SCN. In free running voles ultradian activity bursts were phase locked with CRs (Gerkema et al. 1993). This was confirmed by the analysis of calcium URs in hypothalamic mouse brain slices indicating that URs originated in the SPZ-PVN region which stimulates ultradian activity of the SCN (Wu et al. 2018).

The striatum, a part of the basal ganglia, is a further important player in the expression of URs. Dopamine levels in the striatum of mice, obtained with microdialysis, showed ultradian fluctuations with a period of about 2.5 to 3 h in parallel with URs of locomotor activity. High levels of dopamine correlate with high levels of activity. Knockout of the dopamine transporter lengthened the ultradian activity period from 4 to 14 h (Blum et al 2014). Methamphetamine, which strongly affects brain dopamine levels, prolongs the period of ultradian activity periods in a dose dependent manner, concluding that dopaminergic signalling in the striatum plays a leading role in the expression of URs of rest and activity behaviour (Blum et al 2014; Bourguignon and Storch 2017), suggesting the existence of a **D**opaminergic **U**ltradian **O**scillator (DUO, Blume et al. 2014). At present it is unknown how the striatal dopaminergic signalling and hypothalamic nuclei are interacting with each other to achieve control of systemic URs, as exemplified by the UR*large* in our present study.

Catecholamines play an important role in the expression of brain URs. The release rates of catecholamines and histamine, as well as GABA, glutamate and NO levels fluctuated in an ultradian manner in several brain areas (Philippu et al. 1979; Philippu 2016). These coincided with rhythmic fluctuations of the EEG. Central application of catecholamine and histamine receptor agonists and antagonists modulated the ultradian EEG pattern (Grass et al. 1995; Philippu 2019). Electrocoagulation of the rostral arcuate nucleus abolished ultradian activities suggesting that this brain area plays a central role for brain URs (Grass et al 1996). This underlines the compendious role of ultradian rhythms for general information processing and the state of vigilance of an animal.

### Endocrine control of ultradian rhythms

In our present study we identified two further URs with shorter periods, UR*small* and UR*medium*, which may be linked to endocrine effects on peripheral metabolism. Endocrines which could affect MR in peripheral tissues show a wide range of URs (for review see Grant et al. 2018). Insulin is considered as the major anabolic hormone of the body, responsible for the absorption of glucose from blood into the liver, fat and skeletal muscle and its conversion and storage as glycogen and lipid. Despite its acute response to food intake, it has basal URs with periods of 10 min and 50 min in humans (Simon and Brandenberger 2002) and 5–17 min plus 50–150 min in rats (Chou et al 1994) or 26 min in obese rats (Otukonyong et al. 2005). The short period length of ~ 10 min is probably generated by the pancreatic islands themselves (Chou et al 1994). Similar periodicities were also reported for glucagon, ghrelin and leptin indicating that energy balance is under control of URs with short periods.

The metabolic effects of catecholamines include mobilization of glucose from glycogen stores. Catecholamine levels in the blood of human volunteers changed with an UR of 50–100 min (Schöfl et al 1997). Similar short periods were reported for the release of noradrenaline (54 min), dopamine (37 min) and adrenaline (36 min) from the Locus coerulus of cats (Singewald et al. 1994). In the hypothalamus of rats, pulsatile release rates of noradrenaline (60–120 min, mean 92 ± 4 min), adrenaline (99 min) and dopamine (92 min) could be detected, whereby the releases of adrenaline and noradrenaline coincided to a high extent (Dietl et al. 1993). Elevated levels of peripheral noradrenaline facilitated lipolysis and induced nonshivering thermogenesis in brown adipose tissue causing an immediate rise of MR and T_b_ (Ootsuoka et al. 2009, Blessing et al. 2012).

Glucocorticoids mediate, on a short time scale, glucose availability and lipolysis, and on a long time scale, gene transcription and immune responses. The hypothalamo-pituitary-adrenal axis (HPA) is characterized by a pulsatile release of CRH, ACTH and adrenal release of glucocorticoids (CORT) at about hourly intervals (Jasper and Engeland 1991; Mershon et al. 1992; Spiga et al. 2011; Flynn et al. 2018).

Thyroid hormones affect basal MR and protein biosynthesis. In dairy cows and humans thyroid hormone levels showed URs of about 1.5 h (Bitman et al. 1994). A similar periodicity of pulsatile release of TSH was reported in mares and humans (Buff et al. 2007; Roelfsema et al. 2017) whereas the level of TRH in rats showed an ultradian periodicity of more than 4 h (5.8 pulses in 24 h) (Okauchi et al. 1996) which would be twice as long as the UR period of TSH and the thyroid hormones T4/T3. In a further study on rats TRH and TSH were measured simultaneously (Mizobuchi et al. 1996) confirming this discrepancy, which gave rise to the suggestion that TSH oscillates with double the frequency of TRH (Grant et al. 2018). The evidence for this however is quite small and is based on rat brain microdialysis with hourly sampling intervals, which limits the resolution for measuring URs.

Insulin, catecholamines, CORT, and T4/T3 activate or control metabolism in peripheral tissues on a short time scale between 0.5 and 2 h. Hypothetically they could be involved in the expression of URs. The stable constellation of the three URs even in energetically challenged hamsters, suggests that the endocrine system itself is part of the expression of URs rather than the cause of URs. This underlines the ubiquitous role of URs in control of metabolic processes in cells, tissues and the entire organism. MR is usually considered as a proximate requirement to cover the energy requirements of an animal for thermoregulation and activity. The present findings show that this is only part of the truth. RMR is adjusted to cover the needs of thermoregulation. On top of RMR metabolic bursts are added to cover the needs of activity, behaviour and metabolic processes requiring energy. The latter are under control of endogenous URs which have major effects on total energy balance of an animal to an extent which needs yet to be explored.

## Conclusions

We identified three different ultradian rhythms, instead of only one previously known from Djungarian hamsters and other mammals. They are characterized by different period length and amplitude, URsmall (period ~ 1 h), URmedium (1.5–2.2 h) and URlarge (~ 2.0–5.0 h). Wavelet analysis showed that these URs are present in the time course of metabolic rate, body temperature, and locomotor activity. The three ultradian rhythms are running in parallel and may overlap each other. Their period varies between individuals as well as in each individual from day to day. In long term records the periods of all three URs were observed changing in parallel. This suggests that URs are controlled by separate ultradian oscillators interacting with each other. They are not part of the circadian system, but the lower amplitude of ultradian rhythms during the diurnal resting phase indicates an interaction between circadian rhythms and the expression of ultradian rhythms. Ultradian rhythms of metabolic rate require up to 38% of the daily energy budget. This underlines the significance of rheostasis for control of metabolism, thermoregulation and locomotor activity.

## Abbreviations

CORT: Glucocorticoids and mineralocorticoids
CR: Circadian rhythm
DEE: Daily energy expenditure
DUO: Dopaminergic ultradian oscillator
MR: Metabolic rate (mLO_2_ min^−^^1^)
M_b_: Body mass (g)
PVN: Paraventricular nucleus
RER: Respiratory exchange ratio
RMR: Resting metabolic rate
SCN: Suprachiasmatic nucleus
SPZ: Subparaventricular zone
T_b_: Body temperature (abdominal)
T_sf_: Surface temperature
TSH: Thyroid stimulating hormone
TRH: Thyroid releasing hormone
UR: Ultradian rhythm
UR*small*: Ultradian rhythm with small amplitude
UR*medium*: Ultradian rhythm with medium amplitude
UR*large*: Ultradian rhythm with large amplitude

## References

[CR1] Aschoff J, Gerkema MP (1985) On the diversity and uniformity of ultradian rhythms. Exp Brain Res, Suppl 12:321–334 10.1007/978-3-642-70483-3_21

[CR2] Bitman J, Kahl S, Wood DL, Lefcourt AM (1994) Circadian and ultradian rhythms of plasma thyroid hormone concentrations in lactating dairy cows. Am J Physiol 266:R1797–R1803 8024031 10.1152/ajpregu.1994.266.6.R1797

[CR3] Blessing W, Mohammed M, Ootsuka Y (2012) Heating and eating: brown adipose tissue thermogenesis precedes food ingestion as part of the ultradian basic rest–activity cycle in rats. Physiol Behav 105(4):966–974. 10.1016/j.physbeh.2011.11.009 22115948 10.1016/j.physbeh.2011.11.009

[CR4] Blum ID, Zhu L, Moquin L, Kokoeva MV, Gratton A, Giros B, Storch KF (2014) A highly tunable dopaminergic oscillator generates ultradian rhythms of behavioral arousal. ELife. 10.7554/elife.05105 25546305 10.7554/elife.05105 PMC4337656

[CR5] Borchers HW (2019) pracma: Practical numerical math functions. R Package Version 2(9):519

[CR6] Bourguignon C, Storch KF (2017) Control of rest:activity by a dopaminergic ultradian oscillator and the circadian clock. Front Neurol 8:1–7. 10.3389/fneur:2017.00614 29230188 10.3389/fneur:2017.00614 PMC5711773

[CR7] Braulke LJ, Heldmaier G (2010) Torpor and ultradian rhythms require an intact signalling of the sympathetic nervous system. Cryobiology 60:198–203 19913528 10.1016/j.cryobiol.2009.11.001

[CR8] Braulke LJ, Klingenspor M, DeBarber A, Tobias SC, Grandy DK, Scanlan TS, Heldmaier G (2008) 3-Iodothyronamine: a novel hormone controlling the balance between glucose and lipid utilisation. J Comp Physiol B 178:167–177. 10.1007/s00360-007-0208 17912534 10.1007/s00360-007-0208

[CR9] Brodsky VY (2014) Circahoralian (Ultradian) metabolic rhythms. Biochemistry 79(6):483–495. 10.1134/S0006297914060017 (**Maik Nauka Publishing/Springer SBM**) 25100006 10.1134/S0006297914060017

[CR10] Buff PR, Messer NT 4th, Cogswell AM, Johnson PJ, Keisler DH, Ganjam VK (2007) Seasonal and pulsatile dynamics of thyrotropin and leptin in mares maintained under a constant energy balance. Domest Anim Endocrinol 33(4):430–436. 10.1016/j.domaniend.2006.08.007 17055686 10.1016/j.domaniend.2006.08.007

[CR13] Chou HF, Berman N, Ipp E (1994) Evidence for pancreatic pacemaker for insulin oscillations in low-frequency range. Am J Physiol 266:R1786–1791. 10.1152/ajpregu.1994.266.6.R1786 8024029 10.1152/ajpregu.1994.266.6.R1786

[CR14] Daan S, Aschoff J (1981) Short term rhythms in activity. In: Aschoff J (ed) Biological Rhythms. Plenum Press, NY, pp 491–498

[CR15] Dietl H, Prast H, Philippu A (1993) Pulsatile release of catecholamines in the hypothalamus of conscious rats. Naunyn Schmiedebergs Arch Pharmacol 347(1):28–33. 10.1007/BF00168768 8446181 10.1007/BF00168768

[CR16] Elfers K, Armbrecht Y, Brede M, Mazzuoli-Weber G, Heldmaier G (2022) How much does it cost? Teaching physiology of energy metabolism in mice using an indirect calorimetry system in a practical course for veterinary students. Adv Physiol Educ 46:145–157. 10.1152/advan.00027.2021 34882486 10.1152/advan.00027.2021

[CR17] Flynn BP, Conway-Campbell BL, Lightman SL (2018) The emerging importance of ultradian glucocorticoid rhythms within metabolic pathology. Annales D’endocrinologie 79(3):112–114 29627070 10.1016/j.ando.2018.03.003 PMC5984398

[CR18] Gerkema MP, van der Leest F (1991) Ongoing ultradian activity rhythms in the common vole, Microtus arvalis, during deprivations of food, water and rest. J Comp Physiol A 168:591–597 1920159 10.1007/BF00215081

[CR19] Gerkema MP, Verhulst S (1990a) Warning against an unseen predator: a functional aspect of feeding in the common vole, Microtus arvalis. Anim Behav 40:1169–1178. 10.1016/S0003-3472(05)80183-6 10.1016/S0003-3472(05)80183-6

[CR20] Gerkema MP, Groos GA, Daan S (1990b) Differential elimination of circadian and ultradian rhythmicity by hypothalamic lesions in the common vole. Microtus Arvalis J Biol Rhythms 5(2):81–95. 10.1177/074873049000500201 2133128 10.1177/074873049000500201

[CR21] Gerkema MP, Daan S, Wilbrink M, Hop MW, van der Leest M (1993) Phase control of ultradian feeding rhythms in the common vole (*Microtus arvalis*): The roles of light and the circadian system. J Biol Rhythms 8:151–171 8369551 10.1177/074873049300800205

[CR22] Goh GH, Maloney SK, Mark PJ, Blache D (2019) Episodic ultradian events-ultradian rhythms. Biology 8:15. 10.3390/biology8010015 30875767 10.3390/biology8010015 PMC6466064

[CR23] Grant AD, Wilsterman K, Smarr BL, Kriegsfeld LJ (2018) Evidence for a coupled oscillator model of endocrine ultradian rhythms. J Biol Rhythms 33:475–496. 10.1177/0748730418791423 30132387 10.1177/0748730418791423 PMC6425759

[CR24] Grass K, Prast H, Philippu A (1995) Ultradian rhythm in the delta and theta frequenca bands of the EEG in the posterior hypothalamus of the rat. Neurosci Lett 191:161–164 7644138 10.1016/0304-3940(95)11581-G

[CR25] Grass K, Prast H, Philippu A (1996) Influence of mediobasal hypothalamic lesion and catecholamine receptor antagonists on ultradian rhythm of EEG in the posterior hypothalamus of the rat. Neurosci Lett 207:93–96 8731429 10.1016/0304-3940(96)12494-0

[CR26] Heldmaier G (1975) Metabolic and thermoregulatory responses to heat and cold in the Djungarian hamster, *Phodopus sungorus*. J Comp Physiol 102:115–122 10.1007/BF00691297

[CR27] Heldmaier G, Braulke L, Flick J, Ruf T (2024) Silencing of ultradian rhythms and metabolic depression during spontaneous daily torpor in Djungarian hamsters. J Comp Physiol B. 10.1007/s00360-024-01573-1 38972930 10.1007/s00360-024-01573-1 PMC11316710

[CR28] Honma KI, Hiroshige T (1978) Endogenous ultradian rhythms in rats exposed to prolonged continuous light. Am J Physiol 235(5):R250–R256. 10.1152/ajpregu.1978.235.5.R250 727287 10.1152/ajpregu.1978.235.5.R250

[CR30] Isomura A, Kageyama R (2014) Ultradian oscillations and pulses: coordinating cellular responses and cell fate decisions. Development 141:3627–3636. 10.1242/dev.104497 25249457 10.1242/dev.104497 PMC4197574

[CR31] Jasper MS, Engeland WC (1991) Synchronous ultradian rhythms in adrenocortical secretion detected by microdialysis in awake rats. Am J Physiol 261(5 Pt 2):R1257–1268. 10.1152/ajpregu.1991.261.5.R1257 1951775 10.1152/ajpregu.1991.261.5.R1257

[CR32] Kashiwagi M, Kanuka M, Tanaka K, Fujita M, Nakai A, Tatsuzawa C, Kobayashi K, Ikeda K, Hayash Y (2021) Impaired wakefulness and rapid eye movement sleep in dopamine-deficient mice. Mol Brain 14:170. 10.1186/s13041-021-00879 34794460 10.1186/s13041-021-00879 PMC8600805

[CR33] Leise TL (2013) Wavelet analysis of circadian and ultradian behavioral rhythms. J Circadian Rhyth 11(1):1–9 10.1186/1740-3391-11-5 PMC3717080 23816159

[CR34] Leise TL, Harrington ME (2011) Wavelet-based time series analysis of circadian rhythms. J Biol Rhythms 26(5):454–463 21921299 10.1177/0748730411416330

[CR35] Leise TL, Indic P, Paul MJ, Schwartz WJ (2013) Wavelet meets actogram. J Biol Rhythms 28(1):62–68 23382592 10.1177/0748730412468693 PMC4487858

[CR36] Merson JL, Sehlhorst CS, Rebar RW, Liu JH (1992) Evidence of a corticotropin-releasing hormone pulse generator in the macaque hypothalamus. Endocrinology 130(5):2991–2996 1572307 10.1210/endo.130.5.1572307

[CR37] Meyer CW, Blessing W, Heldmaier G (2012) Ultradian episodes of thermogenesis in mammals: Implications for the timing of torpor entry and arousal. In: Ruf T, Bieber C, Arnold W, Millesi E (eds) Living in a seasonal world. Springer, Berlin, Heidelberg, pp 219–229

[CR38] Mizobuchi M, Ishikawa M, Okauchi Y, Bando H, Saito S (1996) Effects of thyroidectomy on thyrotropin-releasing hormone (TRH) and somatotropin release-inhibiting factor (SRIF) patterns in intrahypophysial microdialysates in rats. Endocr J 43(6):679–687. 10.1507/endocrj.43.679 9075608 10.1507/endocrj.43.679

[CR39] Mohawk JA, Green CB, Takahashi JS (2012) Central and peripheral circadian clocks in mammals. Annu Rev Neurosci 35:445–462. 10.1146/annurev-neuro-060909-153128 22483041 10.1146/annurev-neuro-060909-153128 PMC3710582

[CR40] Nemec AFL, Nemec MJ (1985) A test of significance for periods derived uning phase-dispersion- minimization techniques. Astron J 90(11):2317–2320 10.1086/113936

[CR41] Okauchi Y, Takahashi H, Mizobuchi M, Bando H, Saito S (1996) Thyrotropin-releasing hormone release in normal and hyperthyroid rats as measured by microdialysis. Tokushima J Exp Med 43(3–4):93–100 9100456

[CR42] Ono D, Honma K, Honma S (2015) Circadian and ultradian rhythms of clock gene expression in the suprachiasmatic nucleus of freely moving mice. Sci Rep 5:12310. 10.1038/srep12310 26194231 10.1038/srep12310 PMC4508664

[CR43] Ootsuka Y, de Menezes RC, Zaretsky DV, Alimoradian A, Hunt J, Stefanidis A, Oldfield BJ, Blessing WW (2009) Brown adipose tissue thermogenesis heats brain and body as part of the brain-coordinated ultradian basic rest-activity cycle. Neuroscience 164(2):849–861. 10.1016/j.neuroscience.2009.08.013 19679172 10.1016/j.neuroscience.2009.08.013 PMC2767384

[CR44] Otukonyong EE, Dube MG, Torto R, Kalra PS, Kalra SP (2005) High-fat diet-induced ultradian leptin and insulin hypersecretion are absent in obesity-resistant rats. Obes Res 13:991–999 15976141 10.1038/oby.2005.116

[CR45] Philippu A (2016) Nitric oxide: a universal modulator of brain function. Curr Med Chem 59:2643–2652 10.2174/0929867323666160627120408 27356532

[CR46] Philippu A (2019) Neurotransmitters are released in brain areas according to ultradian rhythms: coincidence with ultradian oscillations of EEG waves. J Chem Neuroanat 96:66–72. 10.1016/j.jchemneu.2018.12.007 30576780 10.1016/j.jchemneu.2018.12.007

[CR47] Philippu A, Dietl H, Sinha JN (1979) In vivo release of endogenous catecholamines in the hypothalamus. Naunyn-Schmiedeberg’s Arch Pharmacol 308:137–142 503245 10.1007/BF00499055

[CR48] Prendergast BJ, Zucker I (2012) Photoperiodic influences on ultradian rhythms of male Siberian hamsters. PLoS ONE. 10.1371/journal.pone.0041723 22848579 10.1371/journal.pone.0041723 PMC3407235

[CR49] Prendergast BJ, Zucker I (2016) Ultradian rhythms in mammalian physiology and behaviour. Curr Opin Neurobiol 40:150–154. 10.1016/j.conb.2016.07.011 27568859 10.1016/j.conb.2016.07.011

[CR50] R Core Team (2022) R: A language and environment for statistical computing. R Foundation for Statistical Computing, Vienna, Austria. https://www.R-project.org/

[CR51] Refinetti R, Kaufman CM, Menaker M (1994) Complete suprachiasmatic lesions eliminate circadian rhythmicity of body temperature and locomotor activity in golden hamsters. J Comp Physiol A 175:223–232 8071897 10.1007/BF00215118

[CR52] Roelfsema F, Boelen A, Kalsbeek A, Fliers E (2017) Regulatory aspects of the human hypothalamus-pituitary-thyroid axis. Best Pract Res Clin Endocrinol Metab 31(5):487–503. 10.1016/j.beem.2017.09.004 29223283 10.1016/j.beem.2017.09.004

[CR53] Roesch A, Schmidbauer H (2018) WaveletComp: Computational Wavelet Analysis. R package version 1.1

[CR54] Ruf T (1999) The Lomb-Scargle periodogram in biological rhythm research: analysis of incomplete and unequally spaced time-series. Biol Rhythm Res 30(2):178–201 10.1076/brhm.30.2.178.1422 11708361

[CR55] Ruf T, Grafl B (2010) Maximum rates of sustained metabolic rate in cold-exposed Djungarian hamsters (Phodopus sungorus): the second wind. J Comp Physiol B 180(7):1089–1098. 10.1007/s00360-010-0476-8 20458591 10.1007/s00360-010-0476-8

[CR56] Ruf T, Stieglitz A, Steinlechner S, Blank JL, Heldmaier G (1993) Cold exposure and food restriction facilitate physiological responses to short photoperiod in Djungarian Hamsters *(Phodopus sungorus)*. J Exp Zool 267:104–112 8409896 10.1002/jez.1402670203

[CR57] Schibler U, Gotic I, Saini C, Gos P, Curie T, Emmenegger Y, Sinturel F, Gosselin P, Gerber A, Fleury-Olela F, Rando G, Demarque M, Franken P (2015) Clock-talk: interactions between central and peripheral Circadian oscillators in mammals. Cold Spring Harb Symp Quant Biol 80:223–232. 10.1101/sqb.2015.80.027490 26683231 10.1101/sqb.2015.80.027490

[CR58] Schöfl C, Becker C, Prank K, von Zur MA, Brabant G (1997) Twenty-four-hour rhythms of plasma catecholamines and their relation to cardiovascular parameters in healthy young men. Eur J Endocrinol 137(6):675–683. 10.1530/eje.0.1370675 9437236 10.1530/eje.0.1370675

[CR59] Simon C, Brandenberger G (2002) Ultradian oscillations of insulin secretion in humans. Diabetes 51(Suppl. 1):258–261 10.2337/diabetes.51.2007.S258 11815489

[CR60] Singewald N, Schneider C, Pfitscher A, Philippu A (1994) In vivo release of catecholamines in the locus coeruleus. Naunyn-Schmiedeberg’s Arch Pharmacol 350:339–345 7845470 10.1007/BF00178948

[CR61] Spiga F, Waite EJ, Liu Y, Kershaw YM, Aguilera G, Lightman SL (2011) ACTH-dependent ultradian rhythm of corticosterone secretion. Endocrinology 152(4):1448–1457. 10.1210/en.2010-1209 21303945 10.1210/en.2010-1209 PMC3060625

[CR62] Stephan FK, Zucker I (1972) Circadian rhythms in drinking behaviour and locomotor activity of rats are eliminated by hypothalamic lesions. PNAS 69:1583–1586 4556464 10.1073/pnas.69.6.1583 PMC426753

[CR64] van Rosmalen L, Hut RA (2021) Negative energy balance enhances ultradian rhythmicity in Spring-programmed voles. J Biol Rhythms 36:359–368. 10.1177/07487304211005640 33878968 10.1177/07487304211005640 PMC8276337

[CR65] Wellbrock AHJ, Eckhardt LRH, Kelsey NA, Heldmaier G, Rozman J, Witte K (2022) Cool birds: first evidence of energy saving nocturnal torpor in free-living common swifts Apus apus resting in their nests. Biol Lett 18:20210675. 10.1098/rsbl.2021.0675 35414223 10.1098/rsbl.2021.0675 PMC9006018

[CR66] Wollnik F, Turek FW (1989) SCN lesions abolish ultradian and circadian components of activity rhythms in LEW/Ztm rats. Am J Physiol 256:R1027–R1039 2785771 10.1152/ajpregu.1989.256.5.R1027

[CR67] Wu YE, Enoki R, Oda Y, Huang ZL, Honma KI, Honma S (2018) Ultradian calcium rhythms in the paraventricular nucleus and subparaventricular zone in the hypothalamus. PNAS 115:E9469–E9478. 10.1073/pnas.1804300115 30228120 10.1073/pnas.1804300115 PMC6176559

[CR68] Yang S, Yamazaki S, Cox KH, Huang YL, Miller EW, Takahashi JS (2022) Coupling-dependent metabolic ultradian rhythms in confluent cells. PNAS 119(45):e2211142119. 10.1073/pnas.2211142119 36322771 10.1073/pnas.2211142119 PMC9659342

